# Realist review of community coalitions and outreach interventions to increase access to primary care for vulnerable populations: a realist review

**DOI:** 10.1186/s13690-023-01105-3

**Published:** 2023-06-24

**Authors:** Vivian Welch, Kevin Pottie, Caroline Gaudet, Micere Thuku, Ryan Mallard, Shannon Spenceley, Nida Amjed, Arpana Wadhwani, Elizabeth Ghogomu, Cathie Scott, Simone Dahrouge, The IMPACT Team

**Affiliations:** 1 Bruyère Research Institute, Methods Centre, 85 Primrose, Ottawa, ON K1R 7G5 Canada; 2 Bruyère Research Institute, C.T. Lamont Primary Health Care Research Centre (CTLC), 85 Primrose, Ottawa, ON K1R 7G5 Canada; 3 University of Calgary, 2500 University Drive NW, Calgary, AB T2N 1N4 Canada; 4 University of Lethbridge, 4401 University Dr W, Lethbridge, AB T1K 6T5 Canada; 5 Lakehead University, 955 Oliver Road, Thunder Bay, ON P7B 5E1 Canada; 6 PolicyWise for Children & Families, #1000, 9925 109 Street, Edmonton, AB T5K 2J8 Canada

**Keywords:** Realist Review, Vulnerable, Coalition, Mobile service, RE-AIM, Primary Health Care

## Abstract

**Background:**

There are meaningful gaps in equitable access to Primary Health Care (PHC), especially for vulnerable populations after widespread reforms in Western countries. The Innovative Models Promoting Access-to-Care Transformation (IMPACT) research program is a Canadian-Australian collaboration that aims to improve access to PHC for vulnerable populations. Relationships were developed with stakeholders in six regions across Canada and Australia where access-related needs could be identified. The most promising interventions would be implemented and tested to address the needs identified. This realist review was conducted to understand how community coalition and outreach (e.g., mobile or pop-up) services improve access for underserved vulnerable residents.

**Objective:**

To inform the development and delivery of an innovative intervention to increase access to PHC for vulnerable populations.

**Methods:**

A realist review was conducted in collaboration with the Local Innovative Partnership (LIP) research team and the IMPACT research members who conducted the review. We performed an initial comprehensive systematic search using MEDLINE, EMBASE, PsycINFO, and the Cochrane Library up to October 19, 2015, and updated it on August 8, 2020. Studies were included if they focused on interventions to improve access to PHC using community coalition, outreach services or mobile delivery methods. We included Randomized Controlled Trials (RCTs), and systematic reviews. Studies were screened by two independent reviewers and the Reach, Effectiveness, Adoption, Implementation, and Maintenance (RE-AIM) framework was used for data extraction and framework analysis to obtain themes. The LIP research team was also allowed to suggest additional papers not included at screening.

**Results:**

We included 43 records, comprising 31 RCTs, 11 systematic reviews, and 1 case control study that was added by the LIP research team. We identified three main themes of PHC interventions to promote access for vulnerable residents, including: 1) tailoring of materials and services decreases barriers to primary health care, 2) services offered where vulnerable populations gather increases the “reach” of the interventions, 3) partnerships and collaborations lead to positive health outcomes. In addition, implementation designs and reporting elements should be considered.

**Conclusion:**

Realist reviews can help guide the development of locally adapted primary health care interventions.

**Supplementary Information:**

The online version contains supplementary material available at 10.1186/s13690-023-01105-3.

## Background

Health Canada defines primary health care as “an approach to health and a spectrum of services beyond the traditional health care system. It includes all services that play a part in health, such as income, housing, education, and environment” [1]. Recent and widespread reforms in Primary Health Care (PHC) in western countries reflect efforts to address the need for PHC to be more inclusive and equitable [2]. Despite these efforts, meaningful gaps in equitable access to PHC remain, especially for vulnerable populations defined as people who are unable to achieve the full potential of their lives because of social and political contextual factors [3, 4]. Poor access to PHC causes unmet healthcare needs and increases healthcare services use [3, 4].

One of the efforts made by Canada and Australia to close the equity-gap in PHC access, was to initiate a joint 5-year research program, IMPACT (Innovative Models Promoting Access-to-Care Transformation) [4]. This program was designed to increase access to PHC for vulnerable populations in three Canadian (Alberta, Ontario, Quebec) and three Australian (Victoria, New South Wales, South Australia) regions and involved stakeholders from Local Innovation Partnerships (LIP)s in the six regions. The LIPs were set up in communities where access-related needs could be identified and addressed by implementing, and testing the most promising interventions to improve PHC access for vulnerable populations [4–6].

Community-based programs have promoted community health and equity [7]. For example, community coalitions and mobile interventions have been effective in improving access to PHC for vulnerable populations [7–9] but there is evidence of poor access to PHC in some Canadian regions [5, 10]. For example, although services such as senior centres, settlement agencies (immigrant services), physician clinics, nursing, pharmacies, social work, youth-serving organizations, family services, schools, and services working with urban Aboriginal populations were available, there were high levels of social and material deprivation among vulnerable Indigenous and immigrant residents in North Lethbridge, Alberta. The IMPACT local innovation partnership (LIP) research team in Alberta, Canada, planned to implement and scale-up a community coalition and outreach health services intervention (i.e., mobile, or pop-up services) to improve access for vulnerable populations.

To inform the design of a local outreach service, we decided to conduct a realist review to understand existing global evidence on how community coalitions and outreach services work in particular contexts to improve access to PHC for vulnerable populations.

### Research questions

The research question was articulated through an iterative dialogue and discussion between the LIP research team and the IMPACT review team, and is as follows*: “How does establishing an outreach services program such as pop-up or mobile service interventions among community and PHC service providers lead to improved access to PHC among vulnerable populations?”.*

Secondary questions were as follows:

- *Q2: How are community organizations working together to enhance outreach services?*
- *Q3: What is the optimal way to implement/provide PHC using outreach services?*
- *Q4: What forms of outreach services are the most approachable and engaging?*
- *Q5: How are community engagement and/or participation best initiated/encouraged?*

## Methods

We used a realist lens approach and based our synthesis methods on two realist reviews [11, 12]. This review was conducted iteratively with the LIP research team. We chose this approach because it was essential to understand the underlying theory to design the planned intervention, by understanding the relationship between the outcomes and the underlying contexts and mechanisms of action of the interventions.

**Fig. 1:**
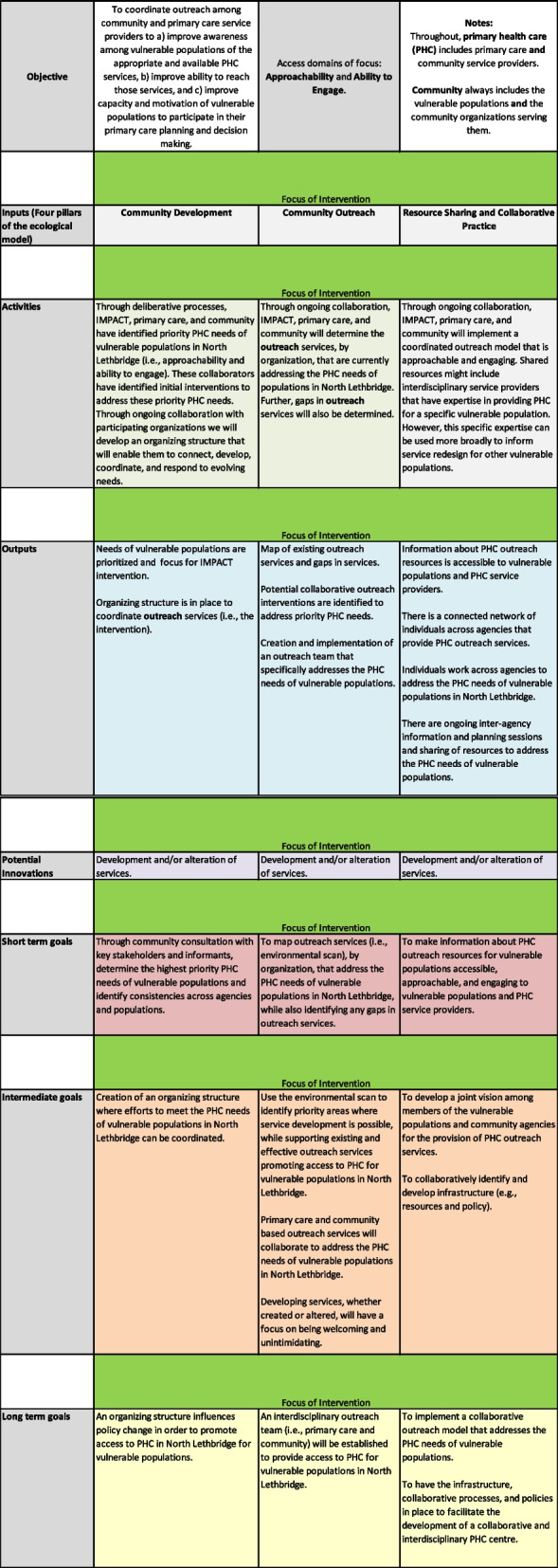
Logic model

We developed a logic model (Fig. 1) in collaboration with the LIP research team, following the Levesque model of access to health care [10] and the W.K. Kellogg Foundation Logic Model Development Guide [13]. This logic model revealed the underlying program theory and assumptions about mechanisms, context, and outcomes. We used this logic model to develop the search strategy.

### Search strategy

The search strategy was developed and tested by an experienced medical information specialist (BS) in consultation with the IMPACT review team and the LIP research team. Using the OVID platform, we searched Ovid MEDLINE®, Ovid MEDLINE® In-Process & Other Non-Indexed Citations, EMBASE, and PsycINFO. We also searched the Cochrane Library on Wiley. Initial searches were performed on October 19th, 2015, and updated on August 8, 2020. Strategies utilized a combination of controlled vocabulary (e.g., “Mobile Health Units”, “Community Networks”, “Vulnerable Populations”) and keywords (e.g., mobile clinics, community partnerships, and at-risk people). See Additional file 1 for the full search strategies used. Vocabulary and syntax were adjusted across databases. Results were limited to the publication years 2005 to August 8, 2020. Filters for RCTs and reviews were applied.

### Study inclusion and exclusion criteria

Studies were eligible if they centred on PHC in combination with at least one community health or social organization or their services. Studies were included if they featured one or a combination of the following elements to target vulnerable populations: mobile service, coalition, outreach, colocation, community development, shared care, pop-up services, resource-sharing, and cooperation. We included RCTs and systematic reviews published in either English or French and set in high-income countries. We excluded duplicates, protocols, dissertations, conference abstracts, qualitative and observational studies, literature reviews, cost-effectiveness analyses or economic evaluations, as well as protocols of studies and reviews. Further, the LIP research team advised the exclusion of studies that did not include some aspect of service provision. The LIP research team was also invited to include up to 5 contextual articles that may not necessarily fit the inclusion criteria.

### Study selection, screening, and abstraction

All retrieved citations from the searches were exported to Covidence. Two independent reviewers (CG, MT, NA, AW, or EG) screened titles and abstracts for eligibility. The full texts of potentially eligible articles were retrieved and screened independently by two reviewers. Disagreements were resolved through discussions. RM and SS from the LIP research team ascertained all included studies for relevance.

A data extraction form based on the RE-AIM framework’s five dimensions (reach, efficacy, adoption, implementation, and maintenance), as well as realist contextual elements, was devised centrally and adapted to the local context in consultation with the LIP research team. The template subsequently incorporated several frameworks: the RE-AIM framework [14–16], the PROGRESS-plus framework [17], and the Template for Intervention Description and Replication (TIDieR) checklist [18]. A complete extraction using this template was done by CG, MT, NA, and AW, and a second extraction focusing on the RE-AIM abstractions was conducted by CG, MT, RM, NA, AW, and SS. When a particular program or intervention was featured in more than one paper (i.e., methodological paper and an outcomes paper), only one paper was chosen as the main publication. Additional data was abstracted from the companion papers.

### Data synthesis

Data synthesis was conducted by CG, NA, AW, in conjunction with RM and SS from the LIP research team. The presence of LIP members ensured that context and local relevancy were considered.

We worked with the LIP research team to identify themes that were relevant to their planned intervention and particular context. After the data was organized and coded according to the RE-AIM framework [14–16], CG, RM, NA, AW, and SS identified patterns in process factors that enhanced or impeded the intervention (Additional file 2 Table 1), as well as patterns in information regarding the theories and mechanisms underpinning the interventions (Additional file 2 Table 2). Outliers were also noted. Finally, the review team discussed and agreed to a set of emerging themes that were then mapped, interpreted, and presented back to the LIP research team for further review and feedback. This process resulted in three themes that are presented below.

**Table 1:** Study characteristics

Study characteristics		Number of included studies
**Quantitative research methods**	Randomized controlled trial (RCT)	31
	Systematic Reviews & Meta-analysis	11
	Case–control	1
**Sample Size**	**RCTs**	
	Range	4407
	Median	330
	Interquartile range (IQR)	644
	**Reviews**^**b**^	
	Range	126
	Median	18
	Interquartile range (IQR)	48
**Types of Intervention**^**a**^	Mobile services	7
	Coalition	24
	Outreach	14
**Duration of intervention**	1–6 months	8
	7–12 months	10
	> 1 year	6
	Varied^c^	10
	No information	9
**Types of providers**	Community health workers (CHWs)	5
	Nurses	2
	Doctors/Pharmacists	4
	Academic staff and community providers	11
	Collaboration between CHWs, nurses, doctors, community members, social worker	18
	Peer support / volunteer	3
**Most common types of outcomes**	Health status	21
	Behavior change	8
	Psychosocial	8
	Access to care	4
	Knowledge translation activities	2
**Countries of intervention**	USA	25
	United Kingdom	4
	Spain	2
	Canada	1
	Multiple countries^d^	9
	No information	2

^**a**^Some reviews assessed multiple interventions

^b^The sample size of reviews is based on the number of included studies

^c^Intervention duration varied for systematic reviews

^d^Some reviews included studies conducted in multiple countries

## Results

**Fig. 2:**
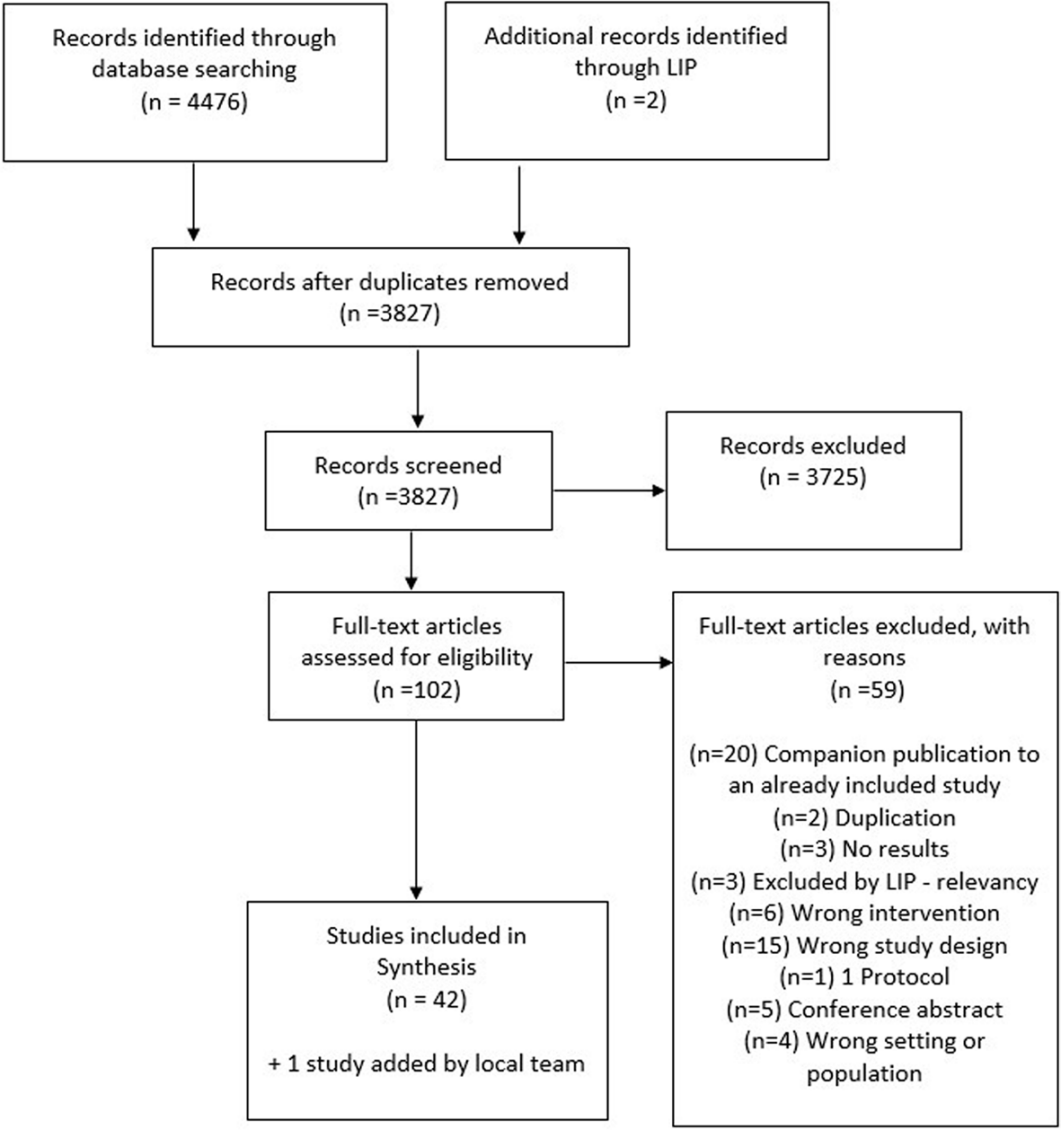
PRISMA flow diagram

We identified 4469 records (2333 through the first search strategy with 10 included) and a total of 42 studies were included in the framework analyses. The LIP research team added one study of their choosing, which was originally excluded due to its study design (case–control), bringing the number of included studies to 43. There were also 20 companion studies, that were considered alongside the main publications. Of the 102 full-text articles assessed for eligibility, 59 were excluded – 20 were companion publications to already included studies, two were duplicates, three did not feature any results, six did not meet intervention eligibility criteria, 15 were of a study design other than review or RCT, one was an RCT protocol, five were conference abstracts and four featured ineligible populations or settings. Three studies were excluded by the LIP team for relevancy. See Fig. 2 for the PRISMA flow chart.

### Description of studies

The characteristics of the 43 included studies are described in Table 1. Additional information can be found in Tables 1 and 2 in Additional file 2, where we present the factors that enhance or impede the intervention, as well as the effects of contextual factors on the intervention. Eleven studies were reviews, 31 were trials, and one study added by the local team was a case–control study. The settings for interventions were predominantly in the community (schools, neighborhoods, homeless shelters, community clinics, mobile health clinics, PHC centres, and churches). Most of the RCTs were conducted in the United States (25 studies). Factors considered to increase vulnerability included racial, ethnic, or minority status (18 studies), low socio-economic status (8 studies), adolescents at risk for drug or alcohol abuse (2 studies), mental health issues (12 studies), elderly population (3 studies), children (5 studies) and being houseless (3 studies). Service providers featured in the studies were from a variety of backgrounds. Coalitions included various members of health and social services, as well as academia, public health, and general community members. The studies reported a range of patient-level, community-level, and service-level outcomes, including health status (22 studies), behavior change (9 studies), various psychosocial outcomes (8 studies), service usage (8 studies), various risk factors (2 studies), quality of life (1 study), community outcomes (1 study), partnership model (1 study), model of care (1 study), and clinic outcomes (1 study).

### Realist analysis and synthesis

Data collection and synthesis were conducted based on the RE-AIM framework. Therefore, the relationship between the outcomes and underlying mechanisms of the included interventions were analysed within the context of the core principles of Reach, Effectiveness, Adoption, Implementation, and Maintenance. We developed themes to explain how the interventions may have improved access to PHC. The included studies in this review demonstrated an overarching emphasis on tailored outreach processes, as well as community-driven intervention designs to elicit higher levels of engagement in and efficacy of the interventions.

### Themes

The three themes identified explain the mechanisms by which awareness and access to PHC services could be improved for vulnerable populations.

#### Tailoring materials and services decreases barriers to PHC for vulnerable populations

Although interventions tended to be complex and included many different components, authors generally endorsed that materials and services should be adapted and tailored to the local context [8, 19–31].

For instance, Hawkins et al. implemented the first community-randomized trial of the Communities that Care (CTC) program [19]. The CTC program is noted as an empowering prevention initiative that incorporates community leaders and a prevention coalition, to discern notable risk and protective factors within the community. A set of interventions designed to reduce the identified elevated risk factors and concurrently encourage the protective factors were then implemented. The authors followed a panel of 4407 students from the fifth grade to the seventh grade in selected communities in seven American states (Colorado, Illinois, Kansas, Maine, Oregon, Utah, and Washington) to explore the outcomes of a CTC program on the reduction of established risk factors, delinquent behaviour, and substance abuse. Community leaders received appropriate training and subsequently identified or established a coalition to administer the CTC process.

There were significantly lower levels of risk factors among students within CTC communities, relative to non-CTC controls 1.67 years after the implementation of the preventative interventions selected through the CTC program. Results further indicated that members of the control group displayed a significantly higher probability of engaging in delinquent behaviour during the study.

This study highlighted the promising effects of community-based partnerships and coalitions on intervention effectiveness and maintenance. The intervention demonstrated potential in the reduction of high-level risk factors within vulnerable groups by adapting the program to the local context.

Chung et al. conducted a study to assess the effects of using depression collaborative care with community engagement and planning (CEP) in minority populations [21]. Implementing the intervention involved tailoring the resources, including the provided depression toolkits to fit the needs of the participating communities. CEP councils, in fact, held bi-weekly meetings for a total of 5 months in order to develop a distinct written training and service delivery plan for each community, using a community engagement model.

Outcomes for this study were based on self-reported surveys conducted at baseline, 6 months and 12 months. Overall, implementing CEP significantly reduced the likelihood of poor-mental health-related quality of life, relative to using traditional resources for service. This study demonstrated the potential of using CEP in depression collaborative care—particularly in underserved communities—considering the limited risks associated with such a model, as well as the positive outcomes pertaining to quality of life.

Other studies, such as O’ Mara Eves et al., and Menon et al., highlight the importance of tailoring key components of interventions to the target demographics [23, 26]. For instance, O’Mara Eves et al., carried out an extensive investigation to evaluate the effectiveness of public health interventions that involve community engagement to address a multitude of health issues [23]. The authors identified that several interventions used media that was personalized to the specific needs of the participants, including newsletters and information sheets. Although there was insufficient evidence to point to one model of community engagement being favourable over the other, it was shown that the interventions had positive impacts on health behaviour outcomes.

Moreover, Menon et al. studied the effectiveness of using a community-to-clinic navigation intervention to enhance colorectal cancer screening among multicultural and underinsured individuals [26]. Participating community sites were randomized to receive group education and reminder calls, or group education and tailored navigation that fit individual concerns and behaviours. Navigators within this study were trained to deduce what may be holding participants back from completing a screening and subsequently provide a tailored message.

Menon et al. found that implementing tailored navigation intervention was associated with an increased rate of cancer screening, with the intervention group being four times more likely to complete screening relative to the control group.

Ultimately, included studies, such as Hawkins et al., Chung et al., O’Mara Eves et al., and Menon et al., highlighted the potential benefits of implementing resources and services to meet individual and/or community-specific needs.

#### Services offered where vulnerable populations gather increases the “reach” of the interventions

Location of services often posed barriers to accessing PHC for vulnerable populations. Two primary methods of countering these barriers are seen in the (30/43) studies included in this review: 1) Services provided in places frequented by the target population [8, 19–22, 24, 27, 30, 32–34], and 2) outreach/mobile clinics that visit where the target population is situated [9, 20, 34–37]. Areas of service provision varied across studies and included schools, social care systems, neighbourhoods, recreational parks, and community organizations. Even when services were offered in locations frequented by the target population, there were sometimes barriers to accessing services. Additional strategies were employed to ensure that the participants reached the services, such as free transportation for those who required it [20, 22, 24], utilising lay health outreach workers to provide the extra nudge to convince and help the vulnerable person seek service [8, 20, 23, 24, 28, 38] or volunteers in each neighbourhood to support the participation of the residents and help them access services [19, 22, 25, 37, 39, 40]. Services were offered at no charge, were covered by insurance or offered at nominal charge [9, 35].

Stagg et al. investigated the efficacy of a peer support intervention in encouraging participation and engagement among marginalized populations, with services targeting chronic Hepatitis C [34]. Participants were identified through outreach services for point-of-care testing for clients with problematic drug use and experiencing houselessness. They were matched with peer advocates who had personally experienced chronic Hepatitis C, allowing them to effectively support others encountering similar challenges. Through peer support, the absolute likelihood of successfully engaging with designated healthcare systems increased by 18.1%, illustrating the positive impact of services through outreach on intervention efficacy.

The MOMmobile was defined as a medical van that provides a range of pre and post pregnancy care at four scheduled locations in urban and rural areas of the Miami-Dade County, South-East Florida, USA [35]. This program resulted in users being significantly more likely to access prenatal care in the first trimester and obtain adequate prenatal care. Services included a wide range of medical and social services, health education, and additional community referral as needed. The MOMmobile services were free for qualified patients, and Medicaid and insurance were accepted for those who were covered.

#### Partnerships and collaborations lead to positive health outcomes for vulnerable populations

Many of the included articles (24/43) had some form of coalition as part of the intervention and their findings were conflicting. Some reviews and RCTs showed that interventions featuring aspects of collaboration, partnership, or community engagement were generally associated with positive outcomes [8, 9, 19–23, 26–28, 32, 34, 41–49], while others showed no difference between interventions and control [25, 50, 51].

Diverse types of partnerships and collaborations included academic-community coalitions, public health – public agency coalitions, and coalitions based on community agency partnerships. The lead sector was most often a university followed by health agency/healthcare provider, not-for-profit community-based organization, community members, and government human service or social welfare agencies. In one systematic review with 58 included studies [8], core group/shared leadership was reported as the predominant coalition leadership type (13/58), followed by steering committee leadership (12/58), single person co-ordinator (3/58), and principal investigator (2/58). Another review [9] reported that the lead academic partner was more often a nursing school than a medical school.

A recurring theme was that coalitions and partnerships required long-term commitment from their members. In Hawkins et al., community leaders were trained for 6–12 months before the implementation of the program, with results that took between 2 to 10 years to be observed [19]. In Redmond et al. article, the delivery of the intervention lasted over four years [32]. In Anderson et al., the average duration of interventions across various types of interventions implemented by coalitions ranged from 20 to 50 months [8].

In the article by Chung some positive outcomes observed at 6 months were not sustained at 12 months, potentially explained by reduced intervention support offered after the first 6 months of implementation [21]. According to Redmond et al., ongoing technical assistance provided by the partnership can result in long-term, high-quality implementation of interventions in the community, which in turn leads to community-level effects [32].

### Implementation design and reporting elements to consider for improving the quality of evidence

Several included studies postulated methods to improve design and reporting of these community-based PHC interventions.

#### Complex versus simple interventions

Most of the interventions tended to be complex in terms of processes, intervention components, as well as partnerships. One systematic review [20] reported that because of the complexity of the interventions, it was unclear which components contributed to the effect of the interventions. The authors proposed that a combination of multiple strategies was more likely to be successful.

#### Theory

All except two of the included studies and reviews [20, 35] disclosed the use of theory for at least some components of the intervention. Various theories were applied in the included studies. The predominant ones included social-ecologic theory, community coalition action theory, community engagement and collaborative care model. Health belief model and social cognitive theory were also seen recurringly. However, theory was generally not discussed at length when presenting and interpreting findings.

#### Adverse outcomes, resource use and costs

In the 43 articles included in this review, adverse outcomes and costs were generally not reported, and when mentioned, were not described in detail. The lack of reporting on adverse outcomes, resource use and costs was also found in one included systematic review [8].

#### Rigorous evaluation

According to Anderson et al., there is a dearth of "rigorous systematic reviews on effectiveness of community coalition models in reducing racial and ethnic disparities in health and well-being.” Evidence is lacking on coalition structures, critical processes, benefits, costs, adverse effects, as well as community contextual factors [8].

### Feedback from local PHC team

The preliminary results of the present review were presented to the LIP research team, as well as to other stakeholders in the design and implementation of the intervention. Feedback was positive, and the themes were all deemed relevant and helpful for improving access to PHC for the vulnerable population in a rural/remote setting in North Lethbridge.

The local team is aiming to achieve themes 1 and 2 by aligning with existing well-attended programs in the community and tailoring the services offered at each pop-up to the local setting, while maintaining a core set of services offered at every pop-up event. For example, when a pop-up service is offered at a seniors’ center, there are regular “core” services offered, such as physician consults, foot care, healthy living programming, dental services, community recreation services, financial planning and supports, and mental health counselling. Tailored services like seniors’ subsidies and benefits counseling, geriatric mental health outreach services, and home care supports are also offered. Tailoring the services, as well as offering the services where the people gather, ensures that services are acceptable and approachable.

Theme 3, “Partnerships and collaborations lead to positive health outcomes for vulnerable populations” is also very relevant and is helpful in guiding the partnerships formed for this project. Unfortunately, within the included studies, we found scant information on the evaluation of partnerships.

Implementation design and reporting elements provided information to keep in mind for successful development and reporting of the intervention. Another important element that was lacking in the literature was whether vulnerable individuals were better connected to family physicians and PHC providers after participating in mobile or pop-up services.

## Discussion

We found that tailoring materials and services, offering the services in regions where vulnerable populations gather, as well as establishing partnerships that include community members and academic partners all contribute to increased access to PHC for vulnerable populations. We also found that studies with multiple strategies led to better outcomes. These strategies should be considered when designing an intervention to improve access for vulnerable populations.

Most interventions were designed based on established theories and the most common was the socio-ecologic theory. The studies do not describe the mechanisms to achieve outcomes, for example, specifics regarding the forms of outreach and optimal ways to provide services.

Nevertheless, the results of this commissioned review were used to design and implement an intervention in North Lethbridge, Alberta. The program evaluation will be published at a later date [6]. This should provide the reader with further information on the utility of a commissioned rapid review in designing and implementing an intervention to increase access to PHC for vulnerable populations.

### Strengths

We followed double, independent selection and extraction processes for all steps of the review to minimize bias in selection of studies. We had a strong partnership with the LIP research team, which informed the logic model and research question as well as interpretation of results.

### Limitations

We modelled our methods based on two previously published rapid realist reviews [11, 12]. In order to limit the scope of the review, we decided to include only systematic reviews and randomized controlled trials. Other types of study designs were included in two different cases: 1) on the recommendation of the LIP research team, and/or 2) if the study was related to a previously accepted randomized controlled trial (companion study). Further expanding the scope to include other study designs, such as qualitative studies, as well as grey literature might identify additional processes to consider and enrich the examples provided here. However, since we included 11 systematic reviews with a total of 348 primary studies, we do have a rich data source for relevant studies.

Another important limitation is the paucity of information available on both mobile clinics and interventions in local settings that offer PHC services tailored to community needs in high-income countries. There is evidence available mainly on low to middle-income countries. Also, we did not search grey literature and websites, thus we may have missed studies and reviews, particularly those led by community partners who may be less likely to publish and those that did not demonstrate effectiveness.

### Generalizability to our local setting and other settings

The results of this realist review have been useful in the efforts to implement a “pop-up” health and social services event in North Lethbridge. Early results demonstrate that this is a promising community-based approach for those who experience barriers to accessing traditional PHC services in North Lethbridge.

There is a need for robust evaluation of mobile and outreach interventions, as well as community coalition service delivery models that focus on health and social services [52]. Luque et al. noted the lack of systematic and rigorous evaluation of mobile clinics, even when mobile clinics obviously lower access barriers [9]. They also cited a lack of generalizability of included studies due to methodological issues. They argued that mobile clinic evaluation reports should include longitudinal data on patient outcomes [9]. There is also a dearth of systematic reviews on effectiveness of community coalitions, as well as insufficient evidence regarding the comparative efficacy of various models of community engagement [8, 23]. In fact, the underlying mechanisms of how these collaborations have a positive effect on outcomes are unclear, with studies generally reporting few details on the structures and processes of these coalitions [8]. One possibility is that coalition-based interventions, particularly when an academic group is involved, are more likely to be evidence-based. Redmond et al. explained that the control group was less likely to use evidence-based interventions, even if they were free to choose from any prevention interventions available [32]. Luque et al. suggested that the addition of partners skilled in program evaluation and research would be beneficial to the coalitions [9].

## Conclusion

This review with a realist lens provides preliminary explanations on the mechanisms involved in mobile and outreach services and collaborative interventions to increase access to PHC for vulnerable populations. Tailoring materials and services, offering the services where citizens experiencing many sources of vulnerability gather, as well as establishing partnerships that include community members and academic partners, all contribute to increased access to PHC for vulnerable populations. The findings of this review support the development, implementation, and evaluation of a “pop up” outreach intervention that is taking place in North Lethbridge, Alberta, which will in turn further inform PHC practice and policy.

## Supplementary Information

**Additional file 1:** Full search strategies.

**Additional file 2: Table 1.** Summary of process factors that enhance or resist the intervention. **Table 2.** Summary of the effects of contextual factors on the intervention.

## Data Availability

All data generated or analysed during this study are included in this published article [and its supplementary information files].

## Abbreviations

CHW: Community health worker
CTC program: Communities that Care program
IMPACT: Innovative Models Promoting Access-to-Care Transformation
LIP: Local innovation partnership
PHC: Primary health care
PROGRESS: Place of residence (urban/rural), Race/ethnicity/culture and language, Occupation, Gender or sex, Religion, Education, Socioeconomic status, Social capital
RCT: Randomized controlled trial
RE-AIM framework: Reach, Effectiveness, Adoption, Implementation, and Maintenance framework
TIDieR checklist: Template for Intervention Description and Replication checklist
