# Supplementary material for: Realist review of community coalitions and outreach interventions to increase access to primary care for vulnerable populations: a realist review
Source: Arch Public Health. 2023 Jun 24;81:115. doi: 10.1186/s13690-023-01105-3 (PMC10290300; doi:10.1186/s13690-023-01105-3)
Supplement: Supplementary file 1 — Additional file 1: Full search strategies. [file 13690_2023_1105_MOESM1_ESM.docx]

Supplementary file

## Additional file 1

### **Search strategies: IMPACT – Alberta**

Update

2020 Aug 8

Ovid Multifile

Database: Embase Classic+Embase <1947 to 2020 August 07> , Ovid MEDLINE(R) ALL <1946 to August 07, 2020>, APA PsycInfo <1806 to August Week 1 2020>

Search Strategy:

--------------------------------------------------------------------------------

1 Mobile Health Units/ (3809)

2 ((mobile or portable or "pop-up") adj2 (care or clinic? or facility or facilities or health or healthcare or service?)).tw,kf. (17133)

3 ((ambulatory or outreach) adj2 (intervention* or service*)).tw,kf. (8172)

4 Community Networks/ (62563)

5 (communit* adj3 (collaborat* or engag* or intervention* or network* or partner* or relations*)).tw,kf. (88279)

6 exp Community Health Services/og (43812)

7 Community-Institutional Relations/ (70932)

8 ((communit* or primary care or organi#ation*) adj3 outreach).tw,kf. (6639)

9 or/1-8 (279906)

10 Vulnerable Populations/ (25172)

11 (("at risk" or deprived or "difficult to find" or "difficult to locate" or "difficult to reach" or disadvantaged or excluded or "hard to find" or "hard to locate" or "hard to reach" or hidden or isolated or low-income or marginal or minority or neglected or underrepresented or under-represented or underresourced or under-resourced or underserved or under-served or unengaged or sensitive or vulnerab*) adj3 (communit* or family or families or neighbourhood? or neighborhood? or patient? or person? or people? or population? or group? or youth?)).tw,kf. (678918)

12 poverty/ (94760)

13 poverty area/ (54199)

14 (deprivation* or (economic* adj1 hardship*) or (economic* adj1 depriv*) or (economic* adj1 disadvantag*) or (financial* adj1 depriv*) or (financial* adj1 disadvantag*) or (financial* adj1 hardship*) or impoverish* or poverty).tw,kf. (283928)

15 Indians, North American/ (34089)

16 ((aboriginal? or native? or indigent or indigenous or inuit?) adj3 (communit* or neighbourhood? or neighborhood? or patient? or person? or people? or population? or group? or youth?)).tw,kf. (69263)

17 Social Stigma/ (29272)

18 (social* adj2 stigma*).tw,kf. (9782)

19 stigmati*.tw,kf. (36155)

20 Shame/ (12229)

21 (shame or shamed or shaming or ashamed).tw,kf. (26041)

22 Social Isolation/ (45490)

23 (social* adj2 (exclud* or exclusion* or inequalit* or inequit* or isolat*)).tw,kf. (50284)

24 Social Marginalization/ (2025)

25 (social* adj2 marginal*).tw,kf. (2570)

26 exp Prejudice/ (40965)

27 (discriminat* or prejudic* or racism).tw,kf. (690193)

28 prisoners/ (44404)

29 prisoner?.tw,kf. (30399)

30 (recent* adj2 release? adj2 (inmate? or prison$ or detainee? or felon? or offender? or convict? or custod* or detention or incarcerat* or correctional or jail? or penitentiar* or prison?)).tw,kf. (450)

31 Medically Uninsured/ (13316)

32 (uninsured* or underinsured* or noninsured or non-insured).tw,kf. (21896)

33 Drug users/ (118568)

34 (drug abus* or drug addict* or drug user?).tw,kf. (141888)

35 exp Substance-Related Disorders/ (532228)

36 Alcoholics/ (135981)

37 (alcoholic? or alcoholism or binge drinking or problem drinking).tw,kf. (276524)

38 ((alcohol or drug? or substance?) adj3 (abus* or addict* or dependen* or delinquency or "illicit use" or misus* or "non-prescription use" or "problem use")).tw,kf. (381841)

39 Prostitution/ (18938)

40 (prostitut* or sex work$3 or sexwork$3 or transactional sex).tw,kf. (28273)

41 Homebound Persons/ (1251)

42 (immobile or (disabled and (house bound or home bound)) or ((house or home) adj3 bound)).tw,kf. (14082)

43 exp Homeless Persons/ (11041)

44 (homeless* or runaway?).tw,kf. (38694)

45 ((destitut* or street) adj3 (bum or bums or individual* or person? or people? or population? or group? or youth?)).tw,kf. (2887)

46 Gypsies/ (1790)

47 (Gypsies or Gypsy or Gipsy or Gipsies or Romany or Romanies or Romani or Romanis or Rromani or Rromanis or Roma).tw,kf. (10109)

48 "Emigrants and Immigrants"/ (17874)

49 "Transients and Migrants"/ (48351)

50 (emigrant? or foreign-born or foreigner? or immigrant? or migrant? or transient?).tw,kf. (824316)

51 Refugees/ (29138)

52 (refugee? or (asylum adj1 seek*)).tw,kf. (34019)

53 (displaced adj3 (child* or family or families or individual* or person? or people? or population? or youth?)).tw,kf. (4782)

54 ((communication* or cultural* or language) adj3 (barrier? or difficult*)).tw,kf. (33457)

55 illiterac*.tw,kf. (5903)

56 or/10-55 (3881297)

57 9 and 56 (44956)

58 systematic review.pt. (132480)

59 exp Systematic Reviews as Topic/ (28783)

60 meta analysis.pt. (118092)

61 exp Meta-Analysis as Topic/ (62349)

62 (meta-analy* or metanaly* or metaanaly* or met analy* or integrative research or integrative review* or integrative overview* or research integration or research overview* or collaborative review*).tw,kf. (461669)

63 (systematic review* or systematic overview* or evidence-based review* or evidence-based overview* or (evidence adj3 (review* or overview*)) or meta-review* or meta-overview* or meta-synthes* or rapid review* or "review of reviews" or technology assessment* or HTA or HTAs).tw,kf. (552404)

64 exp Technology assessment, biomedical/ (25620)

65 (cochrane or health technology assessment or evidence report).jw. (40712)

66 realist review?.tw,kf. (805)

67 or/58-66 (925042)

68 57 and 67 (1236)

69 (controlled clinical trial or randomized controlled trial).pt. (599857)

70 clinical trials as topic.sh. (192448)

71 (randomi#ed or randomly or RCT$1 or placebo*).tw. (2550159)

72 ((singl* or doubl* or trebl* or tripl*) adj (mask* or blind* or dumm*)).tw. (446489)

73 trial.ti. (567344)

74 or/69-73 (3066267)

75 57 and 74 (3929)

76 68 or 75 (4898)

77 exp Animals/ not (exp Animals/ and Humans/) (18013059)

78 76 not 77 (3885)

79 (comment or editorial or news or newspaper article).pt. (2097610)

80 (letter not (letter and randomized controlled trial)).pt. (2217222)

81 78 not (79 or 80) (3876)

82 limit 81 to yr="2005-current" (3266)

83 82 use medall [MEDLINE RECORDS] (1757)

84 ((mobile or portable or "pop-up") adj2 (care or clinic? or facility or facilities or health or healthcare or service?)).tw,kw. (17005)

85 ((ambulatory or outreach) adj2 (intervention* or service*)).tw,kw. (8204)

86 community integration/ (1390)

87 (communit* adj3 (collaborat* or engag* or intervention* or network* or partner* or relations*)).tw,kw. (89008)

88 ((communit* or primary care or organi#ation*) adj3 outreach).tw,kw. (6705)

89 or/84-88 (119453)

90 vulnerable population/ (27938)

91 (("at risk" or deprived or "difficult to find" or "difficult to locate" or "difficult to reach" or disadvantaged or excluded or "hard to find" or "hard to locate" or "hard to reach" or hidden or isolated or low-income or marginal or minority or neglected or underrepresented or under-represented or underresourced or under-resourced or underserved or under-served or unengaged or sensitive or vulnerab*) adj3 (communit* or family or families or neighbourhood? or neighborhood? or patient? or person? or people? or population? or group? or youth?)).tw,kw. (678454)

92 poverty/ (94760)

93 (deprivation* or (economic* adj1 hardship*) or (economic* adj1 depriv*) or (economic* adj1 disadvantag*) or (financial* adj1 depriv*) or (financial* adj1 disadvantag*) or (financial* adj1 hardship*) or impoverish* or poverty).tw,kw. (286556)

94 american indian/ (40763)

95 ((aboriginal? or native? or indigent or indigenous or inuit?) adj3 (communit* or neighbourhood? or neighborhood? or patient? or person? or people? or population? or group? or youth?)).tw,kw. (69363)

96 social stigma/ (29272)

97 (social* adj2 stigma*).tw,kw. (10163)

98 stigmati*.tw,kw. (36358)

99 shame/ (12229)

100 (shame or shamed or shaming or ashamed).tw,kw. (26154)

101 social isolation/ (45490)

102 (social* adj2 (exclud* or exclusion* or inequalit* or inequit* or isolat*)).tw,kw. (51084)

103 exp social exclusion/ (1750)

104 (social* adj2 marginal*).tw,kw. (2628)

105 prejudice/ (34932)

106 (discriminat* or prejudic* or racism).tw,kw. (692022)

107 exp prisoner/ (34942)

108 prisoner?.tw,kw. (30614)

109 (recent* adj2 release? adj2 (inmate? or prison$ or detainee? or felon? or offender? or convict? or custod* or detention or incarcerat* or correctional or jail? or penitentiar* or prison?)).tw,kw. (450)

110 medically uninsured/ (13316)

111 (uninsured* or underinsured* or noninsured or non-insured).tw,kw. (22026)

112 "unlicensed drug use"/ (299)

113 (drug abus* or drug addict* or drug user?).tw,kw. (145267)

114 exp addiction/ (351316)

115 (alcoholic? or alcoholism or binge drinking or problem drinking).tw,kw. (278125)

116 ((alcohol or drug? or substance?) adj3 (abus* or addict* or dependen* or delinquency or "illicit use" or misus* or "non-prescription use" or "problem use")).tw,kw. (385767)

117 prostitution/ (18938)

118 (prostitut* or sex work$3 or sexwork$3 or transactional sex).tw,kw. (28468)

119 homebound patient/ (627)

120 (immobile or (disabled and (house bound or home bound)) or ((house or home) adj3 bound)).tw,kw. (14095)

121 homelessness/ (18925)

122 (homeless* or runaway?).tw,kw. (38937)

123 ((destitut* or street) adj3 (bum or bums or individual* or person? or people? or population? or group? or youth?)).tw,kw. (2894)

124 "romani (people)"/ (570)

125 (Gypsies or Gypsy or Gipsy or Gipsies or Romany or Romanies or Romani or Romanis or Rromani or Rromanis or Roma).tw,kw. (10151)

126 exp migrant/ (50649)

127 immigration/ (55504)

128 (emigrant? or foreign-born or foreigner? or immigrant? or migrant? or transient?).tw,kw. (826333)

129 (refugee? or (asylum adj1 seek*)).tw,kw. (34275)

130 (displaced adj3 (child* or family or families or individual* or person? or people? or population? or youth?)).tw,kw. (4804)

131 ((communication* or cultural* or language) adj3 (barrier? or difficult*)).tw,kw. (33635)

132 illitera*.tw,kw. (14665)

133 or/90-132 (3705067)

134 89 and 133 (28640)

135 limit 134 to (meta analysis or "systematic review") (599)

136 meta-analysis/ (315524)

137 "systematic review"/ (388633)

138 "meta analysis (topic)"/ (42565)

139 (meta-analy* or metanaly* or metaanaly* or met analy* or integrative research or integrative review* or integrative overview* or research integration or research overview* or collaborative review*).kw,tw. (464867)

140 (systematic review* or systematic overview* or evidence-based review* or evidence-based overview* or (evidence adj3 (review* or overview*)) or meta-review* or meta-overview* or meta-synthes* or "review of reviews" or technology assessment* or HTA or HTAs).kw,tw. (555028)

141 biomedical technology assessment/ (24508)

142 (multi* adj treatment* adj2 compar*).kw,tw. (718)

143 (cochrane or health technology assessment or evidence report).jw. (40712)

144 realist review?.tw,kw. (825)

145 or/136-144 (995607)

146 134 and 145 (1060)

147 135 or 146 (1069)

148 randomized controlled trial/ or controlled clinical trial/ (1404230)

149 exp "clinical trial (topic)"/ (328873)

150 (randomi#ed or randomly or RCT$1 or placebo*).kw,tw. (2554565)

151 ((singl* or doubl* or trebl* or tripl*) adj (mask* or blind* or dumm*)).kw,tw. (446762)

152 trial.ti. (567344)

153 or/148-152 (3387603)

154 134 and 153 (3151)

155 147 or 154 (3969)

156 exp animal experimentation/ or exp models animal/ or exp animal experiment/ or nonhuman/ or exp vertebrate/ (52305326)

157 exp human/ or exp human experimentation/ or exp human experiment/ (41174011)

158 156 not 157 (11133106)

159 155 not 158 (3965)

160 editorial.pt. (1199036)

161 letter.pt. not (letter.pt. and randomized controlled trial/) (2211898)

162 159 not (160 or 161) (3961)

163 limit 162 to yr="2005-current" (3580)

164 162 use emczd [EMBASE RECORDS] (1950)

165 ((mobile or portable or "pop-up") adj2 (care or clinic? or facility or facilities or health or healthcare or service?)).tw. (16124)

166 ((ambulatory or outreach) adj2 (intervention* or service*)).tw. (8149)

167 (communit* adj3 (collaborat* or engag* or intervention* or network* or partner* or relations*)).tw. (87639)

168 integrated services/ or outreach programs/ (4898)

169 ((communit* or primary care or organi#ation*) adj3 outreach).tw. (6581)

170 or/165-169 (120157)

171 at risk populations/ (37858)

172 (("at risk" or deprived or "difficult to find" or "difficult to locate" or "difficult to reach" or disadvantaged or excluded or "hard to find" or "hard to locate" or "hard to reach" or hidden or isolated or low-income or marginal or minority or neglected or underrepresented or under-represented or underresourced or under-resourced or underserved or under-served or unengaged or sensitive or vulnerab*) adj3 (communit* or family or families or neighbourhood? or neighborhood? or patient? or person? or people? or population? or group? or youth?)).tw. (676316)

173 Poverty/ (94760)

174 poverty areas/ (54199)

175 disadvantaged/ (18296)

176 (deprivation* or (economic* adj1 hardship*) or (economic* adj1 depriv*) or (economic* adj1 disadvantag*) or (financial* adj1 depriv*) or (financial* adj1 disadvantag*) or (financial* adj1 hardship*) or impoverish* or poverty).tw. (281997)

177 minority groups/ (43026)

178 american indians/ or alaska natives/ or inuit/ (26965)

179 ((aboriginal? or native? or indigent or indigenous or inuit?) adj3 (communit* or neighbourhood? or neighborhood? or patient? or person? or people? or population? or group? or youth?)).tw. (68967)

180 stigma/ (24764)

181 (social* adj2 stigma*).tw. (9596)

182 stigmati*.tw. (36045)

183 shame/ (12229)

184 (shame or shamed or shaming or ashamed).tw. (25983)

185 social isolation/ (45490)

186 (social* adj2 (exclud* or exclusion* or inequalit* or inequit* or isolat*)).tw. (49587)

187 marginalization/ (2318)

188 (social* adj2 marginal*).tw. (2552)

189 exp prejudice/ (40965)

190 (discriminat* or prejudic* or racism).tw. (688079)

191 prisoners/ (44404)

192 prisoner?.tw. (29843)

193 (recent* adj2 release? adj2 (inmate? or prison$ or detainee? or felon? or offender? or convict? or custod* or detention or incarcerat* or correctional or jail? or penitentiar* or prison?)).tw. (450)

194 "uninsured (health insurance)"/ (372)

195 "underinsured (health insurance)"/ (6)

196 Treatment Barriers/ (5068)

197 (uninsured* or underinsured* or noninsured or non-insured).tw. (21832)

198 exp drug abuse/ (446202)

199 exp drug addiction/ (551475)

200 (drug abus* or drug addict* or drug user?).tw. (139927)

201 exp alcoholism/ (239654)

202 (alcoholic? or alcoholism or binge drinking or problem drinking).tw. (272297)

203 ((alcohol or drug? or substance?) adj3 (abus* or addict* or dependen* or delinquency or "illicit use" or misus* or "non-prescription use" or "problem use")).tw. (378241)

204 prostitution/ (18938)

205 (prostitut* or sex work$3 or sexwork$3 or transactional sex).tw. (27977)

206 homebound/ (193)

207 (immobile or (disabled and (house bound or home bound)) or ((house or home) adj3 bound)).tw. (14074)

208 exp homeless/ (7590)

209 (homeless* or runaway?).tw. (38521)

210 ((destitut* or street) adj3 (bum or bums or individual* or person? or people? or population? or group? or youth?)).tw. (2877)

211 romanies/ (1790)

212 (Gypsies or Gypsy or Gipsy or Gipsies or Romany or Romanies or Romani or Romanis or Rromani or Rromanis or Roma).tw. (10081)

213 immigration/ (55504)

214 migrant farm workers/ (438)

215 (emigrant? or foreign-born or foreigner? or immigrant? or migrant? or transient?).tw. (821060)

216 refugees/ (29138)

217 (refugee? or (asylum adj1 seek*)).tw. (33638)

218 (displaced adj3 (child* or family or families or individual* or person? or people? or population? or youth?)).tw. (4759)

219 ((communication* or cultural* or language) adj3 (barrier? or difficult*)).tw. (33317)

220 illitera*.tw. (14595)

221 or/171-220 (3870084)

222 170 and 221 (29702)

223 limit 222 to "0830 systematic review" [Limit not valid in Embase,Ovid MEDLINE(R),Ovid MEDLINE(R) Daily Update,Ovid MEDLINE(R) In-Process,Ovid MEDLINE(R) Publisher; records were retained] (21318)

224 meta analysis/ (315524)

225 (meta-analy* or metanaly* or metaanaly* or met analy* or integrative research or integrative review* or integrative overview* or research integration or research overview* or collaborative review*).tw. (460110)

226 (systematic review* or systematic overview* or evidence-based review* or evidence-based overview* or (evidence adj3 (review* or overview*)) or meta-review* or meta-overview* or meta-synthes* or "review of reviews" or technology assessment* or HTA or HTAs).tw. (548834)

227 realist review?.tw. (787)

228 or/224-227 (873050)

229 222 and 228 (985)

230 223 or 229 (21419)

231 clinical trials/ (105985)

232 (randomi#ed or randomly or RCT$1 or placebo*).tw. (2550159)

233 ((singl* or doubl* or trebl* or tripl*) adj (mask* or blind* or dumm*)).tw. (446489)

234 trial.ti. (567344)

235 or/231-234 (2845440)

236 222 and 235 (3074)

237 230 or 236 (21990)

238 exp Animals/ not (exp Animals/ and Humans/) (18013059)

239 237 not 238 (16787)

240 limit 239 to yr="2005-current" (13529)

241 239 use medall (9501)

242 239 use emczd (6519)

243 240 not (241 or 242) [PSYCINFO RECORDS] (682)

244 83 or 164 or 243 [ALL DATABASES] (4389)

245 remove duplicates from 244 (2860)

246 245 use medall [MEDLINE UNIQUE RECORDS] (1748)

247 (2015101* or 2015102* or 2015103* or 201511* or 201512* or 2016* or 2017* or 2018* or 2019* or 2020*).dt. (6052712)

248 246 and 247 [MEDLINE UNIQUE RECORDS - UPDATE PERIOD] (835)

249 245 use emczd [EMBASE UNIQUE RECORDS] (813)

250 (2015101* or 2015102* or 2015103* or 201511* or 201512* or 2016* or 2017* or 2018* or 2019* or 2020*).dc. (8606634)

251 249 and 250 [EMBASE UNIQUE RECORDS - UPDATE PERIOD] (369)

252 245 not (246 or 249) [PSYCINFO UNIQUE RECORDS] (299)

253 (2015101* or 2015102* or 2015103* or 201511* or 201512* or 2016* or 2017* or 2018* or 2019* or 2020*).up. (41842629)

254 252 and 253 [PSYCINFO UNIQUE RECORDS - UPDATE PERIOD] (124)

255 248 or 251 or 254 [TOTAL UNIQUE RECORDS - ALL DATABASES - UPDATE PERIOD] (1328)

***************************

Cochrane Library

Search Name:

Date Run: 08/08/2020 19:01:37

Comment:

ID Search Hits

#1 [mh "Mobile Health Units"] 64

#2 ((mobile or portable or "pop-up") near/2 (care or clinic or clinics or facility or facilities or health or healthcare or service or services)):ti,ab,kw 1461

#3 ((ambulatory or outreach) near/2 (intervention* or service*)):ti,ab,kw 390

#4 [mh "Community Networks"] 162

#5 (communit* near/3 (collaborat* or engag* or intervention* or network* or partner* or relations*)):ti,ab,kw 5704

#6 [mh "Community Health Services"/OG] 1349

#7 [mh "Community-Institutional Relations"] 183

#8 ((communit* or "primary care" or organi?ation*) near/3 outreach):ti,ab,kw 300

#9 {or #1-#8} 8717

#10 [mh "Vulnerable Populations"] 291

#11 (("at risk" or deprived or "difficult to find" or "difficult to locate" or "difficult to reach" or disadvantaged or excluded or "hard to find" or "hard to locate" or "hard to reach" or hidden or isolated or "low-income" or marginal or minority or neglected or underrepresented or "under-represented" or underresourced or "under-resourced" or underserved or "under-served" or unengaged or sensitive or vulnerab*) near/3 (communit* or family or families or neighbourhood* or neighborhood* or patient or patients or person or persons or people* or population* or group or groups or youth or youths)):ti,ab,kw 67507

#12 [mh Poverty] 1697

#13 [mh "Poverty Area"] 275

#14 (deprivation* or (economic* near/1 hardship*) or (economic* near/1 depriv*) or (economic* near/1 disadvantag*) or (financial* near/1 depriv*) or (financial* near/1 disadvantag*) or (financial* near/1 hardship*) or impoverish* or poverty):ti,ab,kw 8080

#15 [mh "Indians, North American"] 245

#16 ((aboriginal* or native or natives or indigent or indigenous or inuit or inuits) near/3 (communit* or neighbourhood* or neighborhood* or patient or patients or person or persons or people or peoples or population* or group or groups or youth or youths)):ti,ab,kw 954

#17 [mh "Social Stigma"] 227

#18 (social* near/2 stigma*):ti,ab,kw 550

#19 stigmati*:ti,ab,kw 671

#20 [mh Shame] 57

#21 (shame or shamed or shaming or ashamed):ti,ab,kw 401

#22 [mh "Social Isolation"] 286

#23 (social* near/2 (exclud* or exclusion* or inequalit* or inequit* or isolat*)):ti,ab,kw 1082

#24 [mh "Social Marginalization"] 6

#25 (social* near/2 marginal*):ti,ab,kw 29

#26 [mh Prejudice] 347

#27 (discriminat* or prejudic* or racism):ti,ab,kw 9212

#28 [mh Prisoners] 308

#29 prisoner*:ti,ab,kw 660

#30 ((recent* near/2 release*) near/2 (inmate or inmates or prison* or detainee* or felon or felons or offender* or convict or convicts or custody or detention or incarcerat* or correctional or jail or jails or penitentiar* or prison or prisons)):ti,ab,kw 23

#31 [mh "Medically Uninsured"] 66

#32 (uninsured* or underinsured* or noninsured or "non-insured"):ti,ab,kw 384

#33 [mh "Drug users"] 110

#34 ((drug next abus*) or (drug next addict*) or (drug next (user or users))):ti,ab,kw 3950

#35 [mh "Substance-Related Disorders"] 14825

#36 [mh Alcoholics] 9

#37 (alcoholic* or alcoholism or "binge drinking" or "problem drinking"):ti,ab,kw 12527

#38 ((alcohol or drug or drugs or substance or substances) near/3 (abus* or addict* or dependen* or delinquency or "illicit use" or misus* or "non-prescription use" or "problem use")):ti,ab,kw 18803

#39 [mh Prostitution] 97

#40 (prostitut* or (sex next work*) or sexwork* or "transactional sex"):ti,ab,kw 467

#41 [mh "Homebound Persons"] 30

#42 (immobile or (disabled and ("house bound" or "home bound")) or ((house or home) near/3 bound)):ti,ab,kw 213

#43 [mh "Homeless Persons"] 338

#44 (homeless* or runaway*):ti,ab,kw 966

#45 ((destitut* or street) near/3 (bum or bums or individual* or person or persons or people or peoples or population* or group or groups or youth or youths)):ti,ab,kw 30

#46 [mh Gypsies] 2

#47 (Gypsies or Gypsy or Gipsy or Gipsies or Romany or Romanies or Romani or Romanis or Rromani or Rromanis or Roma):ti,ab,kw 127

#48 [mh "Emigrants and Immigrants"] 161

#49 [mh "Transients and Migrants"] 66

#50 (emigrant* or "foreign-born" or foreigner* or immigrant* or migrant* or transient*):ti,ab,kw 17918

#51 [mh Refugee] 107

#52 (refugee* or (asylum near/1 seek*)):ti,ab,kw 444

#53 (displaced near/3 (child* or family or families or individual* or person or persons or people or peoples or population* or youth or youths)):ti,ab,kw 77

#54 ((communication* or cultural* or language) near/3 (barrier* or difficult*)):ti,ab,kw 767

#55 illitera*:ti,ab,kw 345

#56 {or #10-#55} 135401

#57 #9 and #56 Publication Year from 2015 to 2020 26

#58 #9 AND #56 with Cochrane Library publication date Between Oct 2015 and Aug 2020 1526

#59 #57 OR #58 1526

DSR – 12

DSR Protocol – 1

Trials - 1513
