# Supplementary material for: Realist review of community coalitions and outreach interventions to increase access to primary care for vulnerable populations: a realist review
Source: Arch Public Health. 2023 Jun 24;81:115. doi: 10.1186/s13690-023-01105-3 (PMC10290300; doi:10.1186/s13690-023-01105-3)
Supplement: Supplementary file 2 — Additional file 2: Table 1. Summary of process factors that enhance or resist the intervention. Table 2. Summary of the effects of contextual factors on the intervention. [file 13690_2023_1105_MOESM2_ESM.docx]

## Additional file 2

### **Table 1. Summary of process factors that enhance or resist the intervention**

| Authors, Year Type | Focus of intervention | Outcomes | Access factors | Process factors | Participant selection | Settings and approach | Provider Type | Other |
| --- | --- | --- | --- | --- | --- | --- | --- | --- |
| Anderson 2015  Review  (n=58 studies) | Community coalition-driven intervention to reduce health disparities among racial and ethnic minority populations | Community coalitions generally have a positive effect on outcomes; however, underlying mechanisms are unclear Academic - community were the most predominant | Some studies used a lay health outreach worker to increase knowledge, facilitate access to services and promote behavior change | Collaboration across sectors allows pooling of community knowledge and resources while external partners provide financial and technical support.   Establishment of the coalition is a core component of the interventions  Culturally competent interventions | Various racial and ethnic minorities | United States, Australia, Canada, England, Netherlands  Within the studies, various community settings: school, health, mental health and social care systems, neighborhood, recreational parks, community organizations, patient's home | Various providers including community public and private organizations. Three main types of coalitions: 1) grassroots (mainly community), 2) academic institution - community partnership, 3) public health agencies and public agencies. Academic - community were the most predominant | All included studies were deemed at high risk for bias |
| Chung 2014  Other: Ong 2013 Wells 2013    Trial | Two depression collaborative care approaches: 1) Community engagement and planning (CEP) 2)Traditional resource for services (RS) | The CEP intervention reduced the likelihood of poor mental health-related quality of life compared to the RS intervention | Participants were recruited in waiting rooms or at community events | CEP: agencies develop joint strategy and training plan. Tailored depression care toolkits and trainings to each community.  RS model: preset time-limited training to individual agencies including webinars (10 occurred) and site visits (number unknown) for an 8-month period. No tailoring | programs: 133 community agencies and organizations that serve at least 15 persons a week, with at least one staff, and did not focus only on psychotic disorder s or home services  Random assignment  Participants: Mild + depressive symptoms from low-income communities. High dropout rate (41% at 12 months follow-up) | South Los Angeles and Hollywood-Metro communities  Various health and community settings were involved | CEP: Unclear how health providers were utilized. Planning was co-led by community and academic council members  RS: nurse care managers, cognitive-behavioral therapists/psychologists, and psychiatrists. |  |
| Hawkins 2008 Others:Arthur 2010Fagan 2013Fagan 2015Hawkins 2014Hawkins 2009Rhew 2013Van Horn 2014Trial | CoalitionsTailored community prevention system for youth delinquency and substance use | The intervention communities had significantly less delinquent behavior. No significant difference between communities for substance use.Targeted risk factors were also significantly different, control communities had higher risk levels at grade 7 | Programs implemented "where the students are" | Use of Coalition with local leadersUse of an already established system (Communities that Care system) tailored to each community, depending on the chosen risk factors.Use of established evidence-based school-based, community-based youth-focused and family focused programs to address risk factors. | Communities: selected from a pool of matched pairs of communities from 7 states that participated in another prevention study (Community Youth Development Study)Colorado,Illinois, Kansas, Maine, Oregon, Utah, and WashingtonParticipants: Population-based, students in grade 5 in the control and intervention communities.76.1% participation rate. | School, Community, Family | Primary Care providers not explicitly stated. Teachers, human services workers, and community volunteers were trained in the programs and implemented the programs. |  |
| Joshi 2013  Review  (n=25 studies) | Primary Health care delivery models for refugees in resettlement countries | The two main coordination models, case management and team coordination were associated with improved communication and coordination between providers. | Multiple strategies improved access: multidisciplinary staff, use of interpreters, bilingual staff, no cost or low-cost services, outreach services, free transportation, longer clinic opening hours, patient advocacy and gender concordant providers. | Case management approach, by refugee health nurse or other type of health professional most often used to integrate the services. The other main model was team coordination. These lead to improved communication and coordination.  Services should develop models of care according to the needs and the demographics of their local communities. | Refugees in resettlement countries. Various methods of selection. | Resettlement countries: Australia, USA, Switzerland, UK, Canada | Various including: PHC, specialists, allied health, students, volunteers, multidisciplinary teams, network of providers. |  |
| Lu 2012  Review  (n=37 studies) | Cancer screening interventions targeting Asian women | Using multiple strategies appears to be more successful than single interventions  The authors cannot conclude regarding on effectiveness due to heterogeneity of interventions | Various including mobile clinics to provide care where the need is.   Lay health workers, navigators, and home visits helped participants access care in some reported studies.  Free/subsidized services Free transportation Provision of interpreters Provision of female physician | Various, but generally includes culturally tailored care and materials | Asian populations in either home or adopted countries. Various selection processes. | Various locations :US, Taiwan, Thailand, UK, Canada, Singapore, Australia, Thailand, New Zealand, Hong Kong, India Malaysia | Various including educators, health workers, navigators, PCP and more | Because of the variability in interventions reported, the authors cannot conclude what intervention or combination of interventions is most effective.  Authors propose that combination of multiple strategies more likely to be successful than single interventions with Asian women. |
| Luque  2013  Review  (n=18 studies) | Mobile clinics for Migrant and seasonal Farmworkers in the USA | No outcomes, mainly descriptive. | free or nominal charge for the patientsMigrant farmworkers tend to move frequently, tracking and follow-up care difficult at times.The mobile clinics physically go where the need is. Some clinics are year-round. The authors report a missed opportunity regarding men’s health. Activities related to men’s health were usually centered on occupational injuries, other health needs potentially neglected. | Collaborations and partnerships are critical for success and sustainability of these mobile clinics because expenses can be consolidated, culturally appropriate approaches can be developed, duplication of services are reduced, and partners can share risks and rewards. | Mostly Self-selected | The mobile clinics were implemented across various American states.The most common type of clinic was one that travelled to the farm camps. | Variable, but usually an academic partner (i.e., nursing or medical school) led the intervention. Other partners included health departments and organizations specifically focusing on migrant health. | The range of services offered depended on the partners involved and the availability of students and volunteers |
| O'Connel 2009  Case control | MOMmobile, a medical van that travels and provides prenatal and postnatal care.  Includes fridge, fetal monitors, diagnostic equipment, refrigerated medical storage, TV/VCR for education videos  Designed like a traditional health care provider's office. | Users of the MOMmobile were significantly more likely to access prenatal care in the 1st trimester, have adequate prenatal care, and significantly less likely to have preterm births. | MOMmobile visiting 4 scheduled locations in Miami-Dade County  The mothers utilizing the MOMmobile were significantly more likely to access prenatal care in the 1st trimester and have adequate prenatal care.  Address barriers such as language, transportation, and costs | No information | Undocumented immigrants, and the uninsured.  Self-selected. Randomly selected Controls selected from the same population. | MOMmobile visiting 4 scheduled locations in Miami-Dade County | Unspecified PCP | Services include pregnancy test, prenatal postpartum care, Annual Gynecological exams, STI screening, health education, lab work, Women and children eligibility, Healthy Start screenings and community referrals. |
| O'Mara-Eves 2015  Review  (n=131 studies) | Public health interventions that incorporate community engagement strategies. | The interventions had a positive effect on health behavior outcomes, health consequence, health behavior self-efficacy and perceived social support across various conditions.   No sufficient information regarding effectiveness of particular community engagement models. | No information | Most interventions included multiple strategies.  Many interventions contained tailored components (i.e., newsletters and information sheets)  Common strategies: education, advice, social support, skill development training/ | Ethnic minority groups ( mainly "Black". African American or Hispanic/ Latino) , disadvantaged socioeconomic position, and multiple PROGRESS-Plus categorizations | USA, UK, Canada and other OECD countries.  The most common intervention setting was in the community. | Unclear how PCP involved  Interventions were most commonly delivered by peers and by community members. | Close to half of the intervention studies included were deemed at high risk for bias (47.3%) |
| O'Toole 2015  Trial | Outreach intervention that included a personal health assessment and brief intervention, either to 1) Personal health assessment and brief intervention of 2) clinic orientation following personal health assessment | A combination of a personal health assessment and clinic orientation resulted in significant effects. Further, clinical orientations alone resulted in significant effects. Though not significant, personal health assessments alone also resulted in an increase of homeless Veterans access care. The ‘usual care’ control group saw a nominal increase in Veterans accessing care. | The authors site previous work that suggests homeless individuals report not accessing care due to “not knowing where to go for care, previous stigmatizing experience[s] when seeking care, and ‘not caring what happens.’”Transportation to clinic was providedinformation on services and instructions on how to access services as part of usual care. | Personal health assessment and clinic orientation | Homeless veterans not receiving primary care | The research was limited to one geographic region of the United States (i.e., Rhode Island and Massachusetts), an urban setting, and had Veterans health facilities within a 2–3-mile radius.Homeless veterans within a 2-3 mile radius of Veterans Health Administration medical facilities in the USA Participants were recruited from soup kitchens, transitional and emergency shelters, drop in centers, social services agencies. | Social worker, research nurse, research assistant, PHC clinicians | They note that the minimal processes defined in this research (i.e., personal health assessments and clinic orientations) resulted in robust effects, but more intensive intervention (i.e., mobile clinics) might have even more beneficial results. |
| Phillips 2014  Other:  Derges 2014 Phillips 2014b  Trial | Multi component, Community engagement program promoting healthy eating, physical activity, and mental well-being in deprived London neighborhoods | Primary outcomes reported. Primary outcomes were not significantly different in Well London intervention neighborhoods compared with control neighborhoods. However, Intervention neighborhoods had lower unhealthy eating scores and higher proportions agreeing that their community pulled together.  Residents report positive impact in their health and neighborhood experiences. (Derges 2014) | Volunteers and coordinators helped residents access services and improve health behaviors. | All projects were adapted for the local community context. Projects varied for health behavior change activities, local environment modification, cultural activities, and employment and training activities.  Multiple components  Core group of volunteers in each neighborhood | Population level (Neighborhood), however, sampling bias (i.e., cross-sectional interviews)  Low participation rate | UK census lower-super-output-area data was used to determine intervention and control communities in London. The 4 most deprived LSOAs were identified and randomly assigned to either the control or intervention.  Coproduction approach. | No information. Implied that multiple types of providers and organizations were involved | The authors describe “population churn” as being a possible reason for the lack of robust outcomes. Implementing Well London Delivery Teams took longer than expected. |
| Redmond 2009  Other:  Feinberg 2007 Spoth 2007 Spoth 2011 Spoth 2011b Spoth 2013 Spoth 2013b  Trial | Preventive interventions geared towards youth and parents and delivered by Community teams supported by a community-university partnership model "PROSPER" | The intervention has robust significant effects across primary outcomes at the 7th grade assessment, but these effects continually decrease through to the 9th grade assessment. Effect sizes are generally small. However, significant effects generally remain through the 9th grade for protective factors. | Some components delivered in the school, integrated in the curriculum, so all students had access.  Family-focused interventions were offered in community facilities during after-school hours. | There was variation between the sites in the interventions chosen for prevention. For example, some control sites chose similar interventions to experimental sites. | Participants were two cohorts of 6th grade middle school students.  21% drop out rate by 9th grade.  17% of eligible families participated in the family-focused intervention. | 28 Middle schools from rural areas of Iowa and Pennsylvania | University researchers, prevention coordinating teams, and community teams, public school representatives, community human service agency representatives, facilitators. | The control group was more likely to use interventions that were not evidence-based. |
| Rodriguez-Romero 2020  RCT | Community intervention to reduce loneliness in lonely older persons and empower participants to use available resources. | Intervention increased social support, improved perceived mental health and reduced the degree of depression in the intervention group. | 18 sessions consisting of various activities were held over a 6-month period. Patients in intervention group also received weekly phone calls to remind them of day, time, and theme of each session. | Data collected during face-to-face meetings or at PHC center; weekly attendance recorded. Participant satisfaction survey implemented. | Recruitment at urban primary healthcare center by family physicians and nurse practitioners. Aged ≥65 years with moderate or severe perceived loneliness (in accordance with UCLA scale). | Primary healthcare center in central Barcelona. | Intervention led by Nurse Practitioner and involved PHC nurses, family physicians, social workers and neighborhood community agents. |  |
| Petts 2020  Systematic Review | Primary Care interventions used to improve the initiation of mental health services in children. | Effectiveness of interventions to improve mental health service initiation varied considerably. Predominant finding is that engagement interventions were generally no better than control or comparison in improving service initiation. | Various engagement strategies were examined. | 5 studies implemented collaborative intervention, typically involving mental health providers or medical providers; aimed to motivate enrolled participants to follow through with referral. |  |  | Primary care, mental health provider or medical provider. | Note: heterogeneous definitions of service initiation (e.g., accessing online interventions vs. intake for services), as well as different study designs (RCT vs. cohort). |
| Ursua 2018  RCT | CHW intervention to improve hypertension management among Filipino Americans with uncontrolled blood pressure (BP). Assessed efficacy of intervention on BP control, SBP and DBP, and compliance to appointment keeping. | At 8-months, BP controlled among greater percentage of participants in treatment group in comparison to control group (83.3 % and 42.7% respectively), and adjusted odds for controlled BP were 3.2 times the odds of the control group (P < 0.001). Treatment participants showed larger decreases in SBP and DBP, although both groups showed overall decrease in values. | CHWs provided interactive health education by implementing adult learning techniques, and concurrently followed up with participants through individual monthly visits. Follow ups conducted with aim of assisting in linking and negotiating access to a primary care physician, providing social support, and accompanying participants' to scheduled appointments. | Community based participatory research. | Majority (64.9%) women; mean age 53.9 years; 99.6% born outside USA; average residency of 10.3 years in the US. | New York City, USA. All CHWs were Filipino immigrants employed by the study's community partner, Kalusugan Coalition Inc. | One CHW was male, aged 39, and three were female (age range 50–65); all had at least a bachelor's degree. |  |
| Preston 2018  RCT | Community-based intervention, Empowering Communities for Life (EC4L), designed to increase colorectal cancer (CRC) screening through fecal occult blood test (FOBT) in rural underserved communities. | Intervention Arm 2 showed considerably higher CRC screening through FOBT. Community-based participation in conjunction with academic health professionals showed improvement in CRC screening within rural and poorly resourced communities. | CIs reached out to participants themselves; scheduled and held approximately 50 meetings at chosen local sites to enhance attendance. | Bi-monthly meetings as well as training sessions with CIs, CLHWs, and AHPs; to affirm the maintenance of CBPR principles. | 70% African American (AA), 14% White, and 1% other. Race/ethnicity not reported for approximately 15% of enrolled participants. | Two of Arkansas' most medically underserved and poor-resourced communities: Mississippi County and St. Francis County. | Community partners and academic staff. | CIs hired Community Lay Health Workers (CLHWs) in each county and developed a protocol for inviting county residents to a recruitment meeting, followed by an intervention meeting. |
| Kim 2015  RCT | Community-based, culturally tailored, multimodal behavioral intervention program in an ethnic/linguistic minority group with type 2 diabetes. | Intervention and Control group displayed 1.0%–1.3% (10.9–14.2 mmol/mol) and 0.5%– 0.7% (5.5–7.7 mmol/mol) reductions in hemoglobin A1c, respectively. | Structured behavioral education programs via group education; glucose self-monitoring, and individualized motivational counseling with bilingual nurses/CHWs, once a month for 12 months. | Nurses and CHWs had extensive training in DM management. | KA immigrants (average age of 35) with physician diagnosis of DM. | Study took place in USA. A single-center program targeting only one ethnic minority group. | Nurses and CHWs |  |
| Aldridge 2015  RCT | Compare current practice for encouraging tuberculosis screening via mobile digital X-ray unit, among homeless people by volunteer peer educators with direct experience with tuberculosis and/or homelessness. | No evidence to indicate that peer educators increased uptake, as indicated by Poisson Regression; adjusted risk ratio 0.98; 95% CIs 0.80 to 1.20). No adverse events noted. | Peer educators and hostel staff spoke to residents of the hostel, as well as individuals near the hostel location, to encourage screening. | Peer educator volunteers had experience of TB and/or homelessness; recruited via TB clinics in London or from pan-London Find and Treat TB service (F&T). Training provided via a 3-day training session. | 46 hostels participated in study; 2342 eligible residents for screening. | London, UK. Study focused on homeless hostel populations. | Peer educators and volunteers. |  |
| Menon 2019  RCT | Community-to-clinic navigator intervention to guide multicultural, underinsured individuals into primary care clinics to complete colorectal cancer screening. | Tailored navigation intervention for colorectal cancer screening translated into considerable increases in rates of colorectal cancer screening. | Community sites randomized to group education or group education plus tailored navigation, to increase attendance at primary care clinics (Phase I). Individuals who completed a clinic appointment received tailored navigation in person or via phone (Phase II). | Participants attended group education class and continued through intervention according to their assigned group; the comparison group received reminder calls, and the intervention group received tailored navigation calls. | Multicultural, underinsured participants recruited through presentations, flyers, and word of mouth at community centers; 67% female (60 years old on average). 45.2% uninsured; 41.2% receiving Medicare/Medicaid. | Phoenix, Arizona, USA | Primary care and Research staff |  |
| Abbott 2018  RCT | Evaluation of a culturally relevant cardiovascular health promotion intervention that may possibly reduce cardiovascular disease (CVD) risk among a group of rural African American adults. | Demonstrated positive influences on psychosocial variables associated with engaging in cardiovascular health recommendations. Interventions led by Nurses within community settings may also, decrease cardiovascular disease risk. | Health promotion intervention to decrease CVD risk through improvement of intentions, attitudes, norms and self-efficacy. Weekly cardiovascular health promotion education for a total of 6 weeks for intervention group. | A registered nurse educator specializing in CVD prevention and public health delivered the intervention each week in all of the churches. | Predominantly African American congregations. 163 women vs 66 men in the study. Average ages of 59.03 and 56.56 in intervention and control groups respectively. No significant differences in gender, education, and employment status between intervention and control group. | USA, intervention took place in 12 churches in 2 rural counties in northern Florida | Registered nurse |  |
| Berkley-Patton 2020  RCT | Faith Influencing Transformation (FIT) Intervention; a diabetes/CVD screening, prevention and linkage of care pilot intervention; to increase weight loss in African American (AA) church populations. | FIT participants significantly more likely to achieve a > 5 lb. weight loss than controls. | Provided FIT print materials and healthy meals to community members during outreach events; CHLs worked with outreach ministry leaders to motivate participant engagement. Church phone messaging systems encouraged healthy eating/physical activity. | CHLs had 4-hour training sessions before the study and a booster training at 4 months. | Inclusion criteria: self-identified AA, age range between 18-80 years old, ability to complete survey independently in English, not pregnant and no SA. | USA | CHLs, Church ministry leaders and pastors |  |
| Gaughran 2017  RCT | Modular health promotion intervention (IMPaCT Therapy) to improve health and reduce cardiovascular risk in psychosis. | No significant changes associated with physical or mental health component SF-36 scores versus TAU at month 12, or month 15. Additionally, no observed effects for most cardiovascular risk; only HDL cholesterol improved more with IMPACT therapy than TAU (Treatment effect (95% CI); 0.085 (0.007 to 0.16); p = 0.034). | Care coordinators delivered IMPaCT Therapy or TAU alone to their own current patients (cluster). Researchers and the statistician remained blind to treatment allocation. | Community care coordinator received IMPACT training course (4-day). Participating care coordinators were offered fortnightly supervision in IMPaCT Therapy throughout the subsequent 9-month intervention. All care coordinators were offered a one-hour training session for physical health awareness to ensure more standardized TAU. | Eligible participants were as follows: between 18 and 65 years and diagnosed with a psychotic disorder. | Across five mental health NHS trusts in South London, Kent, Sussex, Somerset and Staffordshire, representing an urban to rural population. |  |  |
| Fernandez-Barres 2017  RCT | Nutrition education intervention included in the Home Care Program for caregivers to prevent the increasing risk of malnutrition in dependent, at-risk patients . | Mini Nutritional Assessment test scores of intervention group demonstrated improvements; Group x Time interactions displayed for the following: egg consumption (F=4.1; P=0.018), protein intake (F=3.0; P=0.050), polyunsaturated fatty acid intake (F=5.3; P=0.006), folate (F=3.3; P=0.041) and vitamin E (F=6.4; P=0.002). | Nutrition education for caregivers of 190 dependent patients at risk of malnutrition; within the Home Care Program in various Primary Health Care Centers. | The nurses had previous advanced training in nutrition. Four 2-hour sessions held to standardize the procedure and train the nurses from the various participating Primary Health Care Centers. | Mean age was 87.8 ± 8.9years; more than half (68.2%) women. | Multicenter trial, USA | Nurses and personal caregivers. |  |
| Tsuyuki 2016  RCT | Community pharmacy-based case finding, and intervention focused on cardiovascular risk. | 21% difference (after adjustments) in change in risk for CVD events (p < 0.001) between the intervention and usual care groups. Intervention group showed larger improvements for the following variables: low-density lipoprotein cholesterol (–0.2 mmol/l; p < 0.001), systolic blood pressure (–9.37 mm Hg; p < 0.001), glycosylated hemoglobin (–0.92%; p < 0.001), and smoking cessation (20.2%; p ¼ 0.002). | Medication Therapy Management review from patients' pharmacists and CVD risk assessment and education. Pharmacists prescribed medications and ordered laboratory tests as per their scope of practice to achieve treatment targets. Subjects received monthly follow-up visits for 3 months. | Research team developed an online training program for pharmacists that was reviewed internally and externally for content validity. The training program was hosted online at the Faculty of Pharmacy and Pharmaceutical Sciences, University of Alberta server, and also provided at face-to-face regional meetings. | Mean age was 62; 58% male, 27% smokers. | Alberta, Canada | Pharmacists |  |
| Stanley 2016  RCT | Calmer Life (CL) and Enhanced Community Care interventions delivered by community and expert providers; for preliminary effectiveness on worry, generalized anxiety disorder (GAD) severity, anxiety, depression, sleep, health-related quality of life, and satisfaction. | Demonstrated larger improvement in GAD severity and depression for Calmer Life Intervention participants, in comparison to those receiving Enhanced Community Care. | CL integrated person-centered, flexible skills training to decrease worry; resource counseling to target unmet basic needs; and conduction of communication with primary care providers. Skills training permitted participants to choose session content and number of sessions. | Providers trained via readings, didactics, review of training tapes, role plays, and supervisor review. | Recruitment via self- referral and provider-referral; self-referral included presentations within target communities. | Houston, TX | Community providers, social services |  |
| Cochran 2019  RCT | Brief Motivational Intervention-Medication Therapy Management (BMI-MTM) intervention examined along with its impact on medication misuse and concomitant health conditions. | Initial support provided for the BMI-MTM intervention being acceptable and feasible for delivery, mitigating opioid medication misuse, and improving pain and depression. | Pharmacy based and telephonic sessions to accommodate workflow and time constraints of patients. | The pharmacist component targets medication adherence/misuse, and the subsequent navigator sessions focus on treatment adherence and reduction of psychosocial risk factors. | Eligibility criteria as follows: presently prescribed an opioid medication and screened via community pharmacy sites by one of the designated providers- study pharmacist, pharmacy technician, or research staff. | Southwestern Pennsylvania, USA | Pharmacists |  |
| Reininger 2020  RCT | Community-clinical intervention strategies for a Mexican American population with uncontrolled diabetes; Tested a control program (Salud y Vida 1.0) supporting diabetes management versus an enhanced version (Salud y Vida 2.0) for reductions in HbA1c at 12 months. | At 12 months: Control and Intervention arms both improved HbA1c (mean, (CI), (−0.47 (-0.74 to –0.20)) and (−0.48 (-0.76 to –0.19) respectively). After month 6: intervention group maintained HbA1c levels, but control group HbA1c levels increased. High engagement group demonstrated decreasing pattern throughout period of study; control and lower engagement groups did not sustain HbA1c levels at month 12. | Cross-disciplinary team addressed the individualized needs of the patient beyond basic primary care, such as behavioral health, nutrition, medication compliance, transportation, financial support, and other ancillary services. | Trained university research staff obtained blood to assess Hemoglobin A1c (HbA1c) to conduct a lipid panel. The data collection visits occurred at baseline, 6 months, and 12 months. | Majority female (70.5%, Mexican American (92.1%), and spoke Spanish as their primary language (67.7%). 51.5 years old on average. 61.4% unemployed, 69.4% uninsured and 74.1% made maximum of $1000 a month in their household. 53.2% married, and 59.1% did not graduate from secondary school. | Texas, USA | Healthcare providers, social workers, and other clinic personnel alongside representatives from community-based organizations, including community health workers (CHWs). |  |
| Dodge 2019  RCT | To test implementation and impact of Family Connects (FC) brief universal program to assess family-specific needs, complete brief interventions, and connect families with community resources. Community agencies and families were aligned through an electronic data system. | Nurse home visitation program for families of newborns can be implemented via a community agency with high penetration and quality.  Primary outcome was child protective services investigations for maltreatment. Child abuse investigations revealed a mean (SD) of 0.10 (0.30) investigations for the intervention group vs 0.18 (0.56) investigations for the control group (b = -0.09; 90% CI, -0.01 to -0.12; 95% CI, -0.18 to 0.01; P = .07). | Intervention initiated when a family began within the birthing hospital; followed by 1 to 3 postpartum home visits. Family need for intervention for each of 12 key domains assessed during home visits was further evaluated by designated nurse. | All nurses carrying out the intervention had training. | Selected from a single hospital. | Duke University hospital USA | Trained nurses and community specialist. |  |
| Diez 2018  RCT | Improve contraception use among immigrant and native residents in deprived neighborhoods. | Significant increase in relation to optimal use, within men, women, immigrants and natives in the intervention group; control group displayed no changes. Inconsistent utilization of effective methods decreased by 54.9% and that of less effective methods by 47.2% within intervention group. | A culturally developed and theoretically based brief counselling intervention was delivered in community settings. | All nurses, psychologists and physicians had specific training. Fidelity to the protocol was assessed in the training sessions. The satisfaction of participants was very high. All interviews were performed in community facilities or in a separate facility within the primary health care center. | Women aged 14–49 years and men aged 14–39 years. | Barcelona, Spain | Public health nurses, health psychologist, physicians. |  |
| Hoffman 2018  RCT | Clinic-community partnership model for pediatric obesity treatment. | Higher # of treatment hours (11.4 vs 4.4, SD: 15.3 and 1.6, for intervention and control respectively). Child BMI z score and percent of the 95th percentile at 6 months demonstrated no changes. Larger improvements in physical activity (P = .010) and quality of life (P = .008) for participants within intervention component . | Intervention and control participants both provided with standard clinical care via Healthy Lifestyles. The former concurrently received free, unlimited access to Bull City Fit community-based programming, while the latter was given promotional materials about the local parks and recreation department. | All research staff were trained. | Children characteristics were as follows: 53% female, 51% African American, and 34% Hispanic. Additionally, the mean age was 9.1 years (SD: 1.9); 48% of parents were identified as single, and 26% monolingual in Spanish. | USA | Research assistant | The integrated clinic-community model is a partnership between the Healthy Lifestyles pediatric weight management clinic and a recreation center operated by Durham Parks and Recreation. |
| Meredith 2016  RCT | Compared the effectiveness of minimally enhanced usual care (MEU) versus collaborative care for PTSD with a care manager (PCM). | Similar improvements throughout 1- year evaluation period. PTSD diagnoses displayed absolute decrease of 56.7 % and 60.6 % for PCM and MEU patients respectively, at 12 months; PTSD symptoms decreased by 26.8 (PCM patients)and 24.2 points (MEU patients). No differences in relation to health-associated quality of life. | MEU intervention comprised of PCC education in relation to trauma, PTSD, and evidence-based psychopharmacology. PCM intervention involved the MEU components in conjunction with other elements overseen by designated CMs. | FQHC clinicians trained in relation to evidence-based medication for PTSD (through National Institute for Clinical Excellence guidelines). CMs were also bilingual (English & Spanish) and had prior experience working in FQHC settings. | The average age of 42 among baseline participants; majority of sample was women (80.6 %), as well as a large percentage of Hispanic (51.8 %) and black (35.4 %) patients. | Six FQHCs in New York and New Jersey. | CMs, Clinicians |  |
| Harding 2019  Systematic Review | Investigated the following question: "How are the elements of the He Pikinga Waiora (HPW) Implementation Framework reflected in studies exploring the implementation of a non-communicable disease health intervention in an Indigenous community?" | Studies suggest evidence of following: high community engagement levels, and culture-centeredness. Review conjointly suggests the inhibitions of evidence to practice for long-term duration. | "How are the four elements of the HPW Implementation Framework reflected in studies involving the implementation of a non-communicable disease health intervention in an Indigenous community?" | Two thirds of the interventions included the delivery of at least one component by a community health worker (CHW). | Health conditions investigated were as follows: diabetes, obesity or general non-communicable health conditions, nutrition, and cancer. Intervention types involved lifestyle (38%), multi-pronged, self-management of a condition, and education. | Indigenous communities in Australia, Canada, New Zealand or the United States of America | CHWs | He Pikinga Waiora (HPW) is a recent implementation framework that provides a strong foundation for designing and implementing health interventions in Indigenous communities for non-communicable diseases around community engagement, culture-centered approach, systems thinking and integrated knowledge translation. |
| Schroeder 2018  Systematic Review and Meta-analysis | Community health workers in childhood obesity interventions. | Partnership with community health workers suggested as important strategy in reducing disparities concerning childhood obesity, as well as advancing health equity. | CHWs facilitated health behavior education/ counselling and introduced children and families to the appropriate resources within majority of the designated interventions. Note, CHWs also had varying roles. | CHW training varied greatly in intensity. | Emphasis primarily on children from underserved populations. | Most interventions were delivered in the United States, two studies were based in Auckland, New Zealand and Melbourne, Australia. | CHW were the primary interventionists in five studies, and in others they worked with physicians, nurses, registered dieticians, schools’ staff or clinic staff such as medical assistants. |  |
| Fang 2019  RCT | Community-based educational program to improve knowledge and attitudes toward cervical cancer screening in underserved population. | Overall knowledge regarding cervical cancer and screening guidelines increased (30% for pre-program vs. 88% post- program, p < 0.001); perceived screening benefits also increased from 3.50 vs. 4.49 (p < 0.001); and perceived barriers to screening decreased (3.13 vs. 2.25, p < 0.001). | Intervention participants received one 2-hour educational session via bilingual CHEs. Materials on cervical cancer screening were translated and distributed to participants. | Community leaders and volunteers participated in training sessions focused on re-visitation of project aims and their significance to Vietnamese women, recruitment strategies, and guidelines for administration of the study. | Age ranging from 20 years to 70 years; average age of 52. More than 94% foreign-born with low proficiency in reading or speaking English. | USA | Community leaders and volunteers. | Vietnamese American community leaders were directly involved in the planning, development, and implementation of the project. |
| Pati 2015  RCT | Enriched Medical Home Intervention with home visits from CHWs for child immunization status. | Improvement in newborns and infants/toddlers’ immunization up-to-date status; by more than 15% for children aged 0–2 years old. | CHWs provide support to families; assisted with scheduling appointments, calling the clinician, preparing relevant questions for discussion with clinician, organizing transportation to the appointments, and overseeing the maintenance of health insurance coverage. | CHWs go through matching process; ages, experience, and diverse racial and ethnic backgrounds play prominent roles. CHWs receive 60+ hours of training from clinical staff and the program supervisor. | All pediatric patients at risk for poor health outcomes. | USA; Primary Care Practices providing services to socioeconomically diverse populations. | CHWs; paraprofessionals with minimum of high school education |  |
| Manios 2020  RCT | School and community-based intervention to promote healthy lifestyle, as well as focus on obesity related metabolic risk factors among at risk families. | First year of intervention resulted in improvement regarding certain lifestyle behaviors in high-risk families, within parents. | Primary schools in municipalities used as entry point. | The “all families” component was delivered by the teachers in the intervention schools, who were trained to deliver activities during school hours, to create a more supportive social and physical environment, as well as promote a healthy and active lifestyle. | On the basis of standardized, multi-stage sampling procedure. | Bulgaria, Hungary, Belgium, Finland, Greece and Spain. | Teachers, researchers |  |
| Druss 2017  RCT | Partnership between community mental health center and federally qualified health center in developing a behavioral health home. | Behavioral home associated with considerable improvements in relation to cardiometabolic care and utilization of preventative services. However, majority of clinical outcomes demonstrated no differential improvement between usual care groups and the behavioral health home. | Participants received medical care ON SITE. Care manager provided logistical support to ensure that patients could attend appointments. | Participants received integrated care ON SITE; A treat-to-target approach was used for cardiometabolic risk factors. Both providers attended weekly rounds at community mental health center to facilitate integration of mental health team. | Screened and enrolled using two stage process; identified from list of active patients at community mental health center or referred by mental health providers during the first stage. During Second stage, individuals were screened for abnormalities; must have had a cardiometabolic risk factor. | No information. | Part-time nurse practitioner with prescribing authority and full-time nurse care manager, both supervised by Federally qualified health center’s medical director. |  |
| Lewis 2017  Systematic Review | Evaluating how health care organizations and providers are enabling/enacting integrated care with Indigenous populations as well as the successes and challenges of interventions associated with this population. | Improvement displayed in minimum one of the following areas: physical health, mental health, substance use, and overall wellbeing; 6 articles (from a total of 9) displayed improvements regarding mental health and substance abuse; 3 for physical health; 2 for gains concerning social, vocational, or environmental wellbeing. |  |  | Indigenous patients. | Indigenous serving health care organizations. |  |  |
| Coburn 2012  RCT | Community Based Nursing Intervention by HQP; intervention included integrated, and tightly managed system of care coordination, disease management, and preventive services provided by community-based nurse care managers that worked with primary care providers as well. | HQP Model was shown to decrease all-cause mortality among the designated participants. | Nurse care manager developed an individualized plan for each participant. Nurses also corroborated with the participants’ primary care physicians and specialists, when necessary, to assist enrolled participants in achieving their target clinical goals in conjunction with receiving proper care. | Nurse care managers used a database developed by HQP to track activities and participant contacts, as well as key assessments and clinical data on participants. They also developed individualized plans based on the participant's self-reported primary concerns, risk assessment and evaluation findings, and participant motivational readiness. | Minimum 65 years of age with heart failure, coronary heart disease, asthma, diabetes, hypertension, or hyperlipidemia, and receiving care at a primary care practice agreeing to work with the HQP program. Must also have fee for service Medicare beneficiaries with Parts A and B insurance coverage. | Eastern Pennsylvania, USA. | Nurse Care Managers. |  |
| Vaughan 2017  RCT | Integrating CHWs as part of the team leading a comprehensive diabetes group visit program. Evaluating feasibility of integrating CHWs and examining preliminary evidence of efficacy to improve clinical outcomes and adhere to 8 ADA and USPTF guidelines. | Better clinical results and/or adherence with guidelines within certain areas, including desired A1C levels for intervention group. Control group showed increase in average BMI relative to intervention group which showed a decrease in average BMI. | CHWs assigned to contact participants in- between group visits. | CHWs received Texas State certifications with assistance from authors. Training was in addition to ad hoc access to study physician. | Participants were recruited from a community clinic in southwest Houston with 98% Hispanic patients; (18 or older) with diagnosis type 2 diabetes or pre-diabetes. | Information meeting for participants to inform them of visit structure and obtain consent. | CHWs |  |
| Stagg 2019  RCT | Assess the efficacy of a community-controlled, individual-level, peer support intervention to promote engagement with healthcare services in individuals chronically infected with hepatitis C (HCV). | Peer support may improve patient engagement with healthcare services. | Participants in the intervention arm were individually assigned to a peer advocate from the London-based homeless charity and advocacy organization Groundswell. | Peers have personal experience of a specific illness or lifestyle that enables them to support others experiencing similar challenges. For example, helping individuals to engage with their treatment. | Participants were largely male (278/364, 76.4%), with a median age of 43 years (interquartile range 35–48), born in the UK (276/364, 75.8%) and of White other or White central/eastern European ethnicity (258/364, 76.4%). | London, UK | Peer supporters | Potential participants were approached at outreach services for problematic drug use and homelessness for point-of-care HCV, HBV, and HIV testing |
| Barry 2020  RCT | Bridge Intervention for patients with serious mental illness (SMI) and cancer; feasibility of patient identification, enrollment and study completion; acceptability and apparent benefits. | 23/24 (95.8%) found meeting with the psychiatrist helpful; 16/19 caregivers (84.2%) shared that Bridge addressed key challenges within caregiving. Bridge Delivery was said to be “very” or “most” useful for 94.3% of patients by oncology clinicians. | The intervention team actively communicated with the patient, caregiver, and oncologist, in concurrence with being available for ongoing consultation by phone, text, and page as well. | Intervention team included a psychiatrist with expertise in oncology and CMH, and a case manager within the cancer center. | Participant psychiatric diagnoses included the following: bipolar disorder (50.0%), schizophrenia or schizoaffective disorder (26.7%), and severe major depression (23.3%). 62.1% reported disability benefits as their primary income source, 13.8% lived with a spouse.60% had at least 1 caregiver. | USA; Bridge Intervention Delivery Approach | Psychiatrist, CMH, and a case manager. |  |
| Han 2018  Systematic Review | CHW interventions used in studies varied; included prevention and treatment adherence related self-efficacy, reducing depressive symptoms, quality of life, and adherence motivation. | CHW interventions may have limited efficacy concerning psychosocial outcomes. | Electronic databases used to access RCTs based on inclusion criteria. Access factors within the studies not clearly identified. | Studies used didactic methods as well as role playing to train CHWs. Training length ranged from 2 or 4 half days to 9 days. | Inclusion criteria was as follows; studies were RCTs, tested CHW-led interventions on non-communicable diseases, adult participants with HIV and AIDS. | Studies around the globe were included as well as within community settings. Countries included: U.SA, South Africa, Vietnam and China. | Community Health Workers |  |
| Health 2016  Systematic Review | Reviewed programs that aimed to improve access to primary care practitioners; programs included outreach, orientation, combining health care with other services that homeless people seek, and housing with supportive services | Moderate evidence showing orientation to clinic services alone, or in conjunction with outreach improves accessibility to primary care provider among homeless adults who do not have serious mental illness and currently live in urban centers. |  |  |  | Studies included were conducted in the United States and involved adults who met the homelessness definition/criteria. | A primary care provider (defined as physician, nurse or nurse practitioner). |  |

### **Table 2: Summary of the effects of contextual factors on the intervention**

| Authors and year | Contextual factors | Language and communication | Presence of another program | Theory or mechanism | Other |
| --- | --- | --- | --- | --- | --- |
| Anderson 2015 | Various context. Most included studies targeted individuals in urban settings. | No information | No information | Review based on social-ecologic theory. Individual studies refer to various theories.  The mechanisms of how coalitions help improve health outcomes remain unclear |  |
| Chung 2014  Other: Ong 2013 Wells 2013 | The CEP intervention can be provided over longer term due to the formation of leadership councils and no time constraints. However, the RS intervention is time sensitive and therefore has a beginning and end.  Low SES communities, with high rates of avoidable hospitalizations, low rates of insurance.  Continuing education credit Food | Not reported | Yes, multiple programs. Some in the CEP Intervention, and others in the RS intervention within each of the 2 communities. Potential contamination. | CEP: Community-partnered participatory research  RS: Train the trainer model |  |
| Hawkins 2008  Others: Arthur 2010 Fagan 2013 Fagan 2015 Hawkins 2014 Hawkins 2009 Rhew 2013 Van Horn 2014 | Parent's acceptance required Long term commitment needed on the part of the community leaders (training 6 months-12 months) + implementation of the programs, whose results can be seen 2-10 years later.   These communities have populations ranging from 1500 to 50,000 residents with clear community identities and boundaries. They are small- to moderate-sized towns with their own governmental, educational, and law enforcement structures. | English | No other prevention program targeting prioritized risk factors were present in the intervention and control communities. | CTC’s theory of change. Details provided in the CTC manual. |  |
| Joshi 2013 | Refugees face a lack of knowledge about available services and how they work, language barriers, lack of appropriate services, as well as other psychosocial, sociocultural, political, economic and administrative barriers  Refugees should eventually transition to regular mainstream services | Use of interpreters, bilingual staff | Yes, i.e., partnerships established, and the presence of other health and social programs (i.e., housing) | Accessibility elements by Penchansky and Thomas (1981) |  |
| Lu 2012 | 30% of Asian women have never had a mammogram and 21% have never had a Pap test.  Cultural taboos regarding the topic | Various, some interpreters, others report barriers | No information | No information |  |
| Luque 2013 | Limited funding creating challenges: I.e., expensive diagnostic tests like mammograms | Some clinics used interpreters; others had challenges related to language | Possibly, partnering programs | Keys to sustainability of such programs can be explained by referring to some major constructs from Community Coalition Action Theory |  |
| O'Connel 2009 | Services are free if self-reported clients’ income is less than 200% of the federal poverty line. Medicaid and private insurance were also accepted.  Program was initiated in 1992 after the Hurricane Andrew devastated southern states, impeding access to prenatal care.  Low SES area Fear of deportation  Lack of understanding of US health care system | Authors do not mention how this was addressed | No information |  |  |
| O'Mara-Eves 2015 | Large heterogeneity | Not specified | Unknown | Four different theories of change: 1) Change is facilitated by the community and they mobilize into action 2) need for intervention is identified by observation from outside community, but stakeholder's views are sought through collaboration with community 3) Same as 2) but stakeholder's views are sought through consultation with the community 4) Focus on community engagement in the delivery of the intervention (Lay-delivered) |  |
| O'Toole 2015 | various types of resources were offered to the participants (clothes, food, hygiene kits, benefits representatives) | No information | Yes, multiple programs. These programs were involved in the intervention (soup kitchens, transitional and emergency shelters, drop-in centers) | Behavioral model for vulnerable populations mentioned but need to clarify if this is what was followed |  |
| Phillips 2014 | Low SES neighborhoods Differing priorities between the neighborhoods | No information | Yes, implied. | Community engagement, coproduction, and theory of change |  |
| Redmond 2009 | Population predominantly white and rural. | No information | Control sites were not supported by a Community-University Partnership and therefore were able to use any prevention interventions they deemed fit, whereas experimental sites were guided by Community-University Partnerships that provided technical assistance and guidance at three different levels (i.e., University researchers, prevention coordinating teams, and community teams). | One in class Interventions based on the health belief model, Social learning model, and self-efficacy theory of behavior change. Two interventions based on both the Social learning theory and the problem behavior theory | The authors do not provided details on how long it took to establish Community-University Partnerships. However, the delivery of the interventions was over 4 years (i.e., grades 6-9). The authors hope to publish data on the long-term sustainability of Community-University Partnerships in the future. |
| Rodriguez-Romero 2020 | This intervention could be replicated in other regions, adapting the activities according to neighborhood resources. Collaboration with civic, social and religious centers may be complicated but, as the results show, collaboration is necessary for common goals. | No information | No information | Multidisciplinary activities; corroborating with community agents, social engagement programs, as well as activities enhancing the following: friendship, self-esteem, mental health and well-being. |  |
| Petts 2020 | No Information | No information | No information | Patient engagement model: studies organized and categorized as information provision, patient activation, and patient-provider collaboration. |  |
| Ursua 2018 | Most CHWs lived in Queens, where recruitment also occurred. All CHWs had firm connections to the Filipino community. The CHWs completed a 60-h core-competency training. | All CHWs were Filipino immigrants, fluent in English, Tagalog, and Visayan languages. |  | Health Belief Model and Social Support Theory. |  |
| Preston 2018 | Community partners within Mississippi and St. Francis facilitated training sessions in concurrence with community-capacity development meetings with academic staff to design and implement EC4L (a CBPR study). | No information | No information | Health Behavior Theory | CIs in the targeted counties played an active role in planning and implementing the interventions. CIs devoted more than 80% effort to the project over the five-year study period. |
| Kim 2015 | The counseling team included four RNs and three CHWs. The RNs counseled 38 participants; the CHWs counseled 67 participants. | Bilingual in English and Korean | No information | SHIP-DM was based: Predisposing, Reinforcing, and Enabling Constructs in Education/environmental Diagnosis and Evaluation (PRECEDE)–Policy; Regulatory, and Organizational Constructs in Educational and Environmental Development (PROCEED) model; and self-help model. |  |
| Aldridge 2015 | Peer educators were volunteers, who had experience of TB homelessness or both. | No information | No information | Used volunteer peer educators to improve knowledge. |  |
| Menon 2019 |  | English or Spanish | No information | Tailored navigation, reminder calls |  |
| Abbott 2018 | Church recruitment strategies involved telephone calls to church pastors and representatives, rural church site visits, and referrals from community members. Information about random group placement and the study activities was explained to pastors during the initial contact. | English | No information | Integrated Model of Behavioral Prediction; integrating theoretical aspects of Theory of Reasoned Action, Health Belief Model, and Social Cognitive Theory. |  |
| Berkley-Patton 2020 | AA faith leaders participated in all phases of the study. FIT intervention was constructed to adhere with the strengths of AA churches. | No information | No information | The cultural sensitivity model. |  |
| Gaughran 2017 | Care coordinators in participating CMHTs who were permanently employed and had a minimum of four psychosis patients on their caseload who were eligible to participate in the study. | English | A novel integrated health promotion intervention (HPI), IMPaCT therapy was developed, drawing on key principles of two existing effective interventions the “Well-being Support Programme” and “Managing Mental Health and Drug Use”. | Motivational Interviewing and Cognitive Behavioral Therapy approaches. |  |
| Fernandez-Barres 2017 | The caregivers were informal or formal (relatives of the patients or contracted by them). | Caregivers spoke same language as the patients. | No information | Standardized educational session; based on Spanish recommendations for seniors. | Each of the subjects had a caregiver, and the caregivers were the contents of the nutrition education sessions. |
| Tsuyuki 2016 | Consistent line of communication with family physician of patient following every contact with the patient. Routine follow-up with patients at least every 3-4 weeks for a duration of 3 months. | No information | Communication with patient's family physician. | Broad-based, community pharmacist-initiated vascular risk reduction case-finding and intervention program in patients at high risk for CVD. |  |
| Stanley 2016 | Permitted integration of any R/S beliefs; Participants were allowed to include R/S into any of the modules (core or elective). | English | Other community services | Calmer life and enhanced community care. |  |
| Cochran 2019 | Brief Motivational Intervention-Medication Therapy Management (BMI-MTM) assessed in a single-blinded randomized clinical trial. | English | No information | Pharmacy-based and telephonic sessions set up on appointment basis. |  |
| Reininger 2020 | The SyV 2.0 program works with the system of healthcare in this area, reaching predominantly Mexican American, low-income, underserved individuals with chronic disease rates and related mortality that exceed those in most other regions of the state and the nation. | Spanish, English | SyV 1.0, SyV 2.0 | Developed based on an expanded Wagner’s Chronic Care Management mode. |  |
| Dodge 2019 | "This study tested 5 assertions undergirding the logic of FC: (1) FC can penetrate a large proportion of the birthing community; (2) FC can be delivered with high quality; (3) families assigned to FC will become more connected to community resources; (4) families assigned to FC will demonstrate better parent mental health and parenting; and (5) families assigned to FC will have lower rates of investigation for child maltreatment and better infant and maternal health care participation and outcomes." | No information | No information | Family Connects (FC) program. |  |
| Diez 2018 | Intervention adapted to align with formative investigation data and results. Qualitative study implemented before the development of the program, in accordance with the information presented on reproductive healthcare needs for immigrant women. | No information | No information | Social cognitive theory for counselling sessions. Implementation of intervention followed the Intervention Mapping planning model. |  |
| Hoffman 2018 | Advantages present through integrated model: individualized counseling and treatment, as well as recreation centers that are both local and accessible, provide extended hours, and concurrently have designated staff to give social support to improve family engagement. | Spanish | No information | Clinic-community treatment model incorporated as follows: partnership between an academic medical center and a municipal parks and recreation agency. | All measures were translated into Spanish via an American Translators Association– certified translation service. |
| Meredith 2016 | Objective of the Violence and Stress Assessment (ViStA) study was to explore and determine effectiveness of care management intervention through the context of FQHCs. | Spanish, English | No information | Vista, violence and stress assessment. |  |
| Harding 2019 | No information | English | No information | PRISMA guidelines. |  |
| Schroeder 2018 | Most of the interventions focused on education, support and connection to resources. While 2 interventions focused on upstream environment changes; this included petitioning city council to improve neighborhood parks and working with school partners to improve playgrounds, cafeteria options and school policies. | English | No information | The following were implemented to guide the interventions: Social Cognitive Theory, the Chronic Care Model, Health Belief Model, Transtheoretical Model, behavioral economics, the Warnecke Model for Analysis of Population Health and Health Disparities, the World Health Organization Health Promoting Schools Framework, and the International Obesity Task Force 10 Guiding Principles for Obesity Prevention. |  |
| Fang 2019 | Participants in the control condition received a 2-hour education session delivered by bilingual CHEs. This included topics on general health: healthy lifestyle behaviors and the benefits of obtaining routine medical checkups and cancer screening. | Bilingual, Vietnamese and English | No information | Health Belief Model (HBM) and Social Cognitive Theory (SCT). |  |
| Pati 2015 | CHWs do not administer vaccines or any other medications. CHWs also use tablet computers preconfigured REDCap database. | No information | No information | EMHI (Enriched Medical Home Intervention). |  |
| Manios 2020 | Attempted to provide a more supportive physical environment for all families living in the intervention municipalities; initiatives to encourage an active lifestyle. | No information | No information | Precede-Proceed and HAPA models. |  |
| Druss 2017 | No Information | English | Federally Qualified Health Center and Community Health Center Partnership. | Behavioral Health Home. |  |
| Lewis 2017 | No Information | No information | No information | Integrated care/ multidisciplinary approach. |  |
| Coburn 2012 | The study was designed to evaluate the survival impact of the HQP program versus usual care up to five years post-enrollment. The nurse care managers were community based and worked with patients from multiple primary care practices. | English | Intervention led by HQP was one of the 15 under the MCCD. | Transtheoretical Model of Behavior Change. |  |
| Vaughan 2017 | Authors interviewed potential Hispanic candidates to identify individuals with appropriate personality traits. CHWs recruited from the host site’s pool of bilingual volunteers that work at or live near the clinic. | CHWs were bilingual |  | Data collected based on 8 standards per ADA and USPTF. |  |
| Stagg 2019 | Peer Advocates participated in a six-week training program; program incorporated several modules concerning setting boundaries, safeguarding, governance of information, as well as working with vulnerable adults and blood-borne viruses. New Peer Advocates initially shadowed an experienced Peer. | No information | No information | Peer support mechanism. |  |
| Barry 2020 | Qualitative interviews with oncology and mental health clinicians to obtain thorough understanding of the barriers faced by SMI patients, regarding cancer-care. | English |  | Bridge Intervention Informed by Collaborative Care Model. |  |
| Han 2018 | In describing the CHWs, 10 studies reported using at least one peer (individual living with HIV), and two studies employed lay health workers trained through a local organization, as well as an HIV case manager, a community leader, and a health care worker. | English |  | The systematic review focused on RCTS and conducted a qualitative synthesis of study findings rather than a meta-analysis. |  |
| Health 2016 | A primary care provider was defined as a physician, nurse or nurse practitioner. | English |  | Discussed a logic model that provided a potential framework for how different factors interact to impact access to primary care in people who are homeless, as well as the predicted outcomes. |  |
